# Plasma Membrane Lipid Composition and Turnover in Human Midbrain Neurons Investigated by Time-of-Flight Mass Spectrometry

**DOI:** 10.3390/biom15121650

**Published:** 2025-11-24

**Authors:** Emmanuel Berlin, Alicia A. Lork, Carl Ernst, John S. Fletcher, Nhu T. N. Phan

**Affiliations:** 1 Department of Chemistry and Molecular Biology, University of Gothenburg, 405 30 Gothenburg, Sweden; emmanuel.berlin@gu.se (E.B.); alicia.lork@googlemail.com (A.A.L.); john.fletcher@chem.gu.se (J.S.F.); 2 Montreal Neurological Institute, McGill University, Montreal, QC H3A 0G4, Canada; carl.ernst@mcgill.ca

**Keywords:** plasma membrane, lipid(s), neuron(s), midbrain, lipid turnover, time-of-flight mass spectrometry, secondary ion mass spectrometry imaging

## Abstract

The molecular structure and dynamics of the neuronal plasma membrane are essential for neuronal biology and function. We employed time-of-flight secondary ion mass spectrometry (ToF-SIMS) imaging to investigate the lipid composition and turnover at the plasma membrane of single human midbrain neurons. The results showed that the profile of lipid turnover was heavily influenced by the types of precursors incorporated into the membrane lipids. In addition, there was a high prevalence of phosphatidylcholines, phosphatidylserines, and ceramides in the human midbrain neurons, and a preference for incorporating stearic acid into membrane lipids compared to other precursors. These features indicate a direct link between the membrane lipids to the biological state and functions of midbrain neurons. This is among a very few studies using mass spectrometry imaging to provide an insight into the native membrane lipid organization and lipid turnover using various lipid precursors in human neurons at a single cell level, illustrating their biological relevance in neuronal functions.

## 1. Introduction

The midbrain is a central part of the brain that plays a key role in motor control and processing visual and auditory information [1]. Among different types of brain cells in the midbrain are dopaminergic, GABAergic, and glutaminergic neurons [2,3]. Plasma membrane lipids have been shown to influence the membrane curvature, which affects the synaptic activity and neurotransmitter secretion, signal transduction, and lipid homeostasis [4,5,6]. The loss of neurons in the midbrain is mostly associated with the neurodegenerative disease known as Parkinson’s disease, or disorders such as restless legs syndrome, attention deficit hyperactivity disorder (ADHD), and schizophrenia [7,8,9,10]. Due to the complexity of the midbrain’s structures and functions, it is essential to further understand the molecular organization of the plasma membrane of midbrain neurons.

The plasma membrane in neurons is a highly organized and dynamic structure supporting cellular shape and structures (e.g., movement, budding, cell division, tubulation, synapses, axons, and dendrites), intracellular trafficking, extra-and intracellular signaling, exo- and endocytosis, protein interactions, vesicular trafficking, and cell differentiation, etc. [11,12]. To maintain cellular function, the lipid composition of the plasma membrane is dynamically adapted and renewed via a turnover process, in which old lipid molecules are replaced by newly synthesized ones. Different types of lipids and their relative compositions have significant effects during cell differentiation, cell signaling, and intracellular processes [4,12,13,14,15,16]. The composition and turnover of plasma membrane lipids were found to be altered in the aforementioned diseases, which implies that they are closely related to the cell status and neuronal activity [17,18,19,20,21,22,23,24,25,26,27,28,29,30,30,31,32].

Despite the evident roles in neuronal functions, the lipid composition and turnover of the plasma membrane in human neuronal cells remain largely elusive. This could be explained by the limited availability of neuronal cells from humans. The development and differentiation procedures from human induced pluripotent stem cells (iPSCs) into specific neuronal cell types have been significantly improved [33,34], but expertise is critically needed in one’s lab for successful cell culture. In addition, single-cell analysis techniques providing specific molecular structural information of intact cellular membranes have not been established until more recently, with the emergence of gas cluster ion beams (GCIBs) using Time-of-flight secondary ion mass spectrometry (ToF-SIMS) [35,36,37,38]. Liquid chromatography mass spectrometry (LC-MS) has been the most prominent method to analyze the lipid composition and turnover of cells. However, it is constrained by a limitation to analyze the plasma membrane composition in a bulk solution of cell lysates. Other methods have been developed to selectively analyze plasma membrane lipids, such as the employment of colloidal silica beads, but they could disrupt the cells, and the specificity of the isolated plasma membrane lipids cannot be guaranteed [39].

ToF-SIMS is capable of analyzing plasma membrane lipids at a single-cell level without cell disruptions. The cells can be cultured on a conductive surface (e.g., silicon wafers or indium tin oxide-coated glass), followed by quick rinsing and plunge-freezing to preserve the plasma membrane integrity [40,41]. Membrane lipids have also been studied using supported lipid bilayer models for chemical modeling with TOF-SIMS [42,43,44,45]. Upon ToF-SIMS analysis, a primary ion beam is rastered over the sample surface, which ejects the molecules from the sample as secondary ions. Sample molecules typically up to 3000 Da. can be detected in parallel, depending on the instrumental setup. ToF-SIMS presents some limitations, particularly sensitivity (ppm), which is a trade-off with the imaging resolution. Mass resolution is limited to around 10 000, depending on the detected mass, and sample charging may occur in low-conducting samples [46,47,48,49,50]. The invention of the GCIBs has substantially increased the applications of ToF-SIMS in biology, especially research on lipid membrane structure [35,36,37,38].

We employed ToF-SIMS imaging and a shotgun approach to investigate the lipid composition and turnover of the plasma membrane in single human midbrain neurons. The major lipid composition in the native plasma membrane was identified. Different fatty acids and lipid headgroups were used as lipid precursors to examine their turnover effects. We aim to provide an insight into the biological relevance of the identified membrane lipids that support future research in neuronal functions and diseases in human cells.

## 2. Materials and Methods

### 2.1. Cell Culture and Preparation

Human neuronal progenitor cells (NPCs) were obtained from Carl Ernst’s lab at McGill University, Montreal. The use of these human cells was approved by the Research Ethics Board of the McGill University Health Center (Montreal, QC, Canada) with the ethics approval code 23-09-075 and the date of approval of 9 December 2024. NPCs were thawed rapidly in a water bath at 37 °C and transferred into a T25 flask (Fisher Scientific, Nunc™ EasYFlask™, #156340, Gothenburg, Sweden) coated with poly-D-lysine (Sigma-Aldrich, #P7280, Solna, Sweden) and laminin (Sigma-Aldrich, #L2020, Stockholm, Sweden). The NPCs were cultured until confluent in NPC medium (STEMCELL technologies, STEMDiff Neural Progenitor Basal Medium, #05834, Grenoble, France) containing 200 ng/mL sonic hedgehog (Genescript, #Z03067, Piscataway, NJ, USA).

Indium tin oxide (ITO) glasses (Bruker Nordic, #8237001, Solna, Sweden) previously coated with poly-ornithine (Sigma-Aldrich, #P3655, Solna, Sweden) and laminin were placed in a 24-well plate (Fisher Scientific, #353047, Gothenburg, Sweden) containing the cell culture medium. The NPCs were subsequently transferred onto the ITO glass slides and allowed to adhere in the incubator. After one day, the medium was replaced with midbrain differentiation medium (BrainPhys (STEMCELL technologies, #05790, Saint Egrève, France) supplemented with 2% B27 (Fisher Scientific, #17504044, Gothenburg, Sweden), 1% N2 (Fischer Scientific, #A1370701, Gothenburg, Sweden), 20 ng/mL brain-derived neurotrophic factor (BDNF, Genescript, #Z03208, Piscataway, NJ, USA), 20 ng/mL glial cell line-derived neurotrophic factor (GDNF, Genescript, #Z03387, NJ, USA), 200 nM ascorbic acid (STEMCELL technologies, Cat. #72132, Saint Egrève, France), 1 mM dibutyl cAMP (STEMCELL technologies, Cat. #100-0244, Saint Egrève, France), 1 µg/mL laminin). Half of the cell medium was replaced every 2–3 days for 2 weeks until the cells became fully matured midbrain neurons (5 weeks) and were then incubated with lipid precursors. Two biological replicates were incubated with each isotopically (^13^C-, or ^15^N-) labeled lipid precursor at a final concentration of 50 μM. The examined ^13^C lipid precursors were ^13^C-lauric acid (Eurisotop, #CLM-1586, Saint-Aubin, France), ^13^C-stearic acid (Eurisotop, #CLM-490-1, Saint-Aubin, France), ^13^C-linoleic acid (Eurisotop, #CLM-6855-0.25, Saint-Aubin, France), ^13^C-linolenic acid (Sigma-Aldrich, #694940, Solna, Sweden), ^13^C_2_-ethanolamine chloride (Sigma-Aldrich, #E6133-100G, Solna, Sweden), and ^15^N-choline chloride (Sigma-Aldrich, #609269, Solna, Sweden). The cell cultures were replaced with half of the medium containing isotopic lipid precursor every second day for 4 days. Control cell samples were also prepared under the same experimental conditions without incubation with isotopic lipid precursor. After 4 days of isotopic incubation, the cell cultures were incubated in the medium without isotopic lipid precursors for 12 h. Subsequently, the cells were quickly rinsed four times with prewarmed ammonium formate solution at 150 mM (Sigma-Aldrich, #70221-25G-F, Solna, Sweden) and once with prewarmed milliQ-water to remove excess salts from the cell medium, followed by snap freezing in cold 2-methylbutane (Sigma-Aldrich, #270342-1L, Solna, Sweden) at −185 °C. The cells were stored in liquid nitrogen until they were freeze-dried at a high vacuum of 0.05 × 10^−3^ mbar (Martin Christ, Alpha 1–2 LDplus, Osterode am Harz, Germany) before ToF-SIMS measurements.

### 2.2. Immunocytochemistry

To validate the differentiation of the NPCs into mature midbrain neurons, the cells were stained for microtubule-associated protein 2 (MAP2) and tyrosine hydroxylase (TH), which are two markers associated with mature midbrain neurons. MAP2- and TH-staining was performed on NPCs and differentiated midbrain neurons by incubating cells grown on cover slips first in a staining buffer containing PBS (Sigma-Aldrich, #P4417, Solna, Sweden), 1% bovine serum albumin (Sigma-Aldrich, #A2153, Solna, Sweden), and 0.1% Triton-X100 (Sigma-Aldrich, #T8787, Solna, Sweden) for 1 h. Next, the cells were incubated with MAP2 rabbit primary antibody (Abcam, #ab32454, Cambridge, UK) and TH mouse primary antibody (Abcam, #ab129991, Cambridge, UK) at a 1:200 dilution in the staining buffer for 1 h. The samples were then washed 3 times in PBS and followed by a similar incubation procedure with an anti-rabbit 580 nm secondary antibody (Abberior, #ST580-1002-500UG, Goettingen, Germany) for MAP2 and an anti-mouse 638 nm secondary antibody (Abberior, STRED-1001-500UG, Goettingen, Germany) for TH-labeling. After incubation, the samples were washed once with PBS, stained for 5 min in 1:1000 DAPI (nucleus staining, Sigma-Aldrich, #D9542, Solna, Sweden) diluted in PBS, washed twice with PBS before being mounted (Abberior, #MM-2011-2X15ML, Goettingen, Germany) on microscopic slides for imaging.

The samples were fluorescently imaged using the Abberior Expert Line microscope (Abberior, Goettingen, Germany) in confocal mode with a 100× UPLSAPO NA 1.4 oil immersion objective (Olympus, Tokyo, Japan). MAP2 was imaged at an excitation wavelength of 561 nm and an emission spectrum of 575–630 nm, while TH was imaged at an excitation wavelength of 640 nm and an emission spectrum of 650–763 nm. DAPI was imaged with an emission spectrum of 415–583 nm. All samples were imaged with 10% laser intensity at 1 AU, a dwell time of 15 µs, an image size of 78 × 78 µm, and a pixel size of 100 nm. MAP2 and TH were imaged using 3-line acquisitions, while DAPI was imaged with a 1-line acquisition. The image intensities of TH and MAP2 were then normalized based on the background signal at 8-bit, whilst the DAPI signal was set freely using Fiji, version 1.54p [51]. The verification can be observed in Figure S1, in which the midbrain neurons have a higher expression of MAP2 and TH compared to undifferentiated NPCs.

### 2.3. ToF-SIMS Imaging

A J105—3D Chemical Imager ToF-SIMS instrument (Ionoptika Ltd., Southampton, UK) equipped with a 40 keV (CO_2_)_6000_^+^ primary gas cluster ion beam (GCIB) was used for sample analysis [52,53]. Samples were analyzed in both positive and negative ion modes with a mass range from 100 to 1000 Da. Before imaging, a cell area of 600 × 600 µm^2^ was rastered for surface cleaning with 64 × 64 pixels and a primary ion dose of 3 × 10^12^ ion/cm^2^. Sequentially, the same cell area was imaged with a primary ion current of 15 pA, 696 shots/pixel, at either 128 × 128 pixels with a primary ion dose of ∼1.1 × 10^13^ ion/cm^2^, or at 256 × 256 pixels with a primary ion dose of ∼4.7 × 10^13^ ion/cm^2^. Two biological replicates were obtained for each sample condition, and 3 to 5 cell areas were imaged for each replicate (*n* is 6–10 for each sample condition).

### 2.4. Data Analysis

SIMS data was extracted using the Ionoptika Image Analyzer software, version 3.0.0 (Ionoptika Ltd., Southampton, UK) by selecting cell areas identified via the PC-headgroup ion signal (*m*/*z* 184.07) in positive mode, and FA 16:0 and FA 18:0 (*m*/*z* 255.23 and 283.25, respectively) in negative mode, examples shown in Supplementary data Figure S2. After selecting cell areas, the spectral data was exported and consecutively binned to 0.05 Da in Matlab version R2021a (The Mathworks Inc., Gothenburg, Sweden), resulting in a mass accuracy of ~65 ppm at *m*/*z* 800. Major interference peaks from the ITO glass substrate were then removed from the spectral data, and the peak intensities were normalized to the total ion intensity of all the mass peaks of the ROIs within a SIMS image.

Lipid class abundance was detected using ChiToolbox in Matlab with the code adapted from previous literature [54,55] to detect the 90 most highly abundant lipids (~5% of the total peak number), 45 of the most abundant peaks in each ion mode.

Next, the lipid profiles of the cellular plasma membrane after incubation with different isotopic lipid precursors, including ^13^C_2_-ethanolamine and ^15^N-choline, ^13^C-linoleic acid and ^13^C-linolenic acid, ^13^C-linoleic acid and ^13^C-stearic acid, ^13^C-linolenic acid and ^13^C-stearic acid, and ^13^C-lauric acid and ^13^C-stearic acid, were compared. Lipid turnover was evaluated via the isotopic incorporation based on the changes in the peak intensity across the entire mass spectrum of the treated samples compared to those of the control. This was obtained using principal components analysis (PCA) in SIMCA, version 17.0.0.24543 (Sartorius, Goettingen, Germany) to select the mass peaks contributing to the distinct differences between the isotopically incubated samples and the control. The selected peaks were then analyzed statistically using GraphPad Prism, version 10.3.0 (GraphPad Software, MA, USA). All comparisons were performed using a multiple Mann–Whitney test with a *p*-value of ≤0.05.

The significant mass peaks were sequentially assigned using the LMSD database LIPIDMAPS^®^ (https://www.lipidmaps.org/, accessed on 9 July 2024) and with peaks detected in positive mode in the typical ion forms of [M+H]^+^, [M+H–H_2_O]^+^, [M+Na]^+^, or [M+K]^+^, while typical ions of [M−H]^−^ were detected in negative mode. A peak assignment was given with an accuracy of no higher than 100 ppm. Detection of ceramides, phosphatidylglycerol, and phosphatidylethanolamine was based on previous studies [12,53,56,57,58,59,60,61,62,63,64,65,66] as well as lipid fragment generation [12,16,67,68,69,70,71,72,73,74]. The lipid assignment list can be found in the Supplementary Materials, Tables S1 and S2.

## 3. Results and Discussion

### 3.1. Lipid Abundancy in Neuronal Plasma Membrane

**Figure 1:**
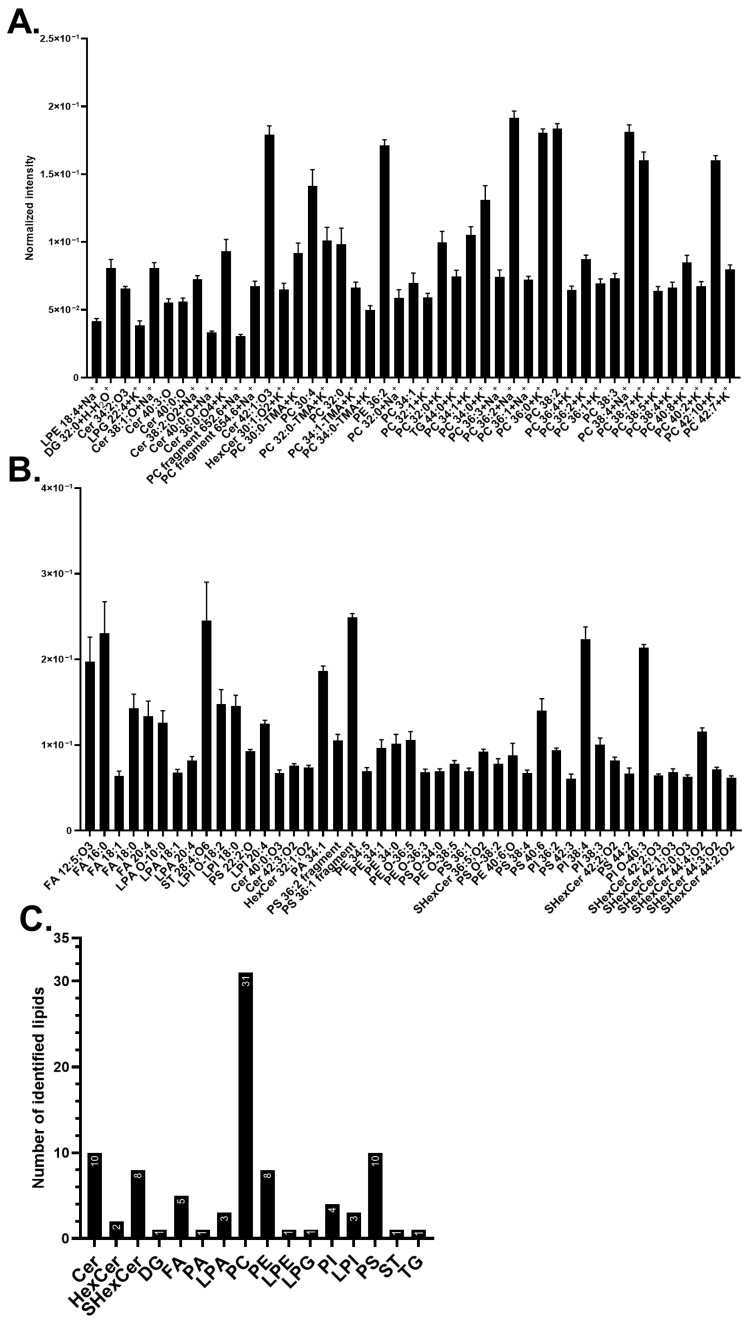
Major membrane lipid compositions of human midbrain neurons. (**A**) The 45 most abundant lipids detected in positive mode. (**B**) The 45 most abundant lipids detected in negative mode. The lipid names on the x-axis from left to right are in ascending mass/charge (*m*/*z*). Error bars are in the standard error of the mean (SEM). (**C**) Summary of the abundant lipid classes detected in positive (**A**) and negative (**B**) modes. The analysis was performed on five biological replicates with 20 measurements in total. The lipid classes included: ceramide (Cer), diacylglycerol (DG), fatty acid (FA), hexosylceramide (HexCer), lysophosphatidic acid (LPA), lysophosphatidylethanolamine (LPE), lysophosphatidylglycerol (LPG), lysophosphatidylinositol (LPI), phosphatidic acid (PA), phosphatidylcholine (PC), phosphatidylethanolamine (PE), phosphatidylinositol (PI), and phosphatidylserine (PS), sulfatide (SHexCer), sterol (ST), and triacylglycerol (TG). Lipids with no annotated ionization were detected as [M+H]^+^ in (**A**) and [M–H]^−^ in (**B**).

Human midbrain neurons were first analyzed to detect the native abundance of major lipid components in the plasma membrane. Figure 1A,B displays the abundance of the 45 most common lipids detected in each ion mode. This includes 35 different lipids in positive mode, where some lipids were detected in different ionization forms such as molecular ions, salt adducts, and fragments, and 44 unique compounds in negative mode. The number of lipids in each lipid class is shown in Figure 1C.

Phosphatidylcholine (PC) species were found to be the most abundant lipid (Figure 1A,C); their salt adducts and fragments have the total carbon numbers ranging from C_32_ to C_42_ and 0 to 8 double bonds. Phosphatidylserine (PS) species are the second most common lipid class (Figure 1B,C) along with ceramides (Cers). PSs have carbon chains ranging from C_22_ to C_44_ and 0 to 6 double bonds, whilst Cers are composed of carbon chains from C_34_ to C_42_ with 0 to 3 double bonds. Among those, compounds [PC 38:2+H]^+^ at *m*/*z* 814.63, [PC 36:2+Na]^+^ at *m*/*z* 808.58, and [PC 38:4+Na]^+^ at *m*/*z* 832.58 are the three most abundant lipids in positive mode (Figure 1A) whereas PS fragment [PS 36:1–87-H]^−^ at *m*/*z* 701.51, sterol (ST) [ST 28:4;O_6_-H]^−^ at *m*/*z* 473.29, and fatty acid (FA) [FA 16:0-H]^−^ at *m*/*z* 255.23 are the three most abundant in negative mode (Figure 1B). The occurrence of different lipid classes amongst highly abundant lipids is summarized in Figure 1C, where the three major membrane components are PCs (~33%), PSs (~11%), and Cers (~11%), followed by sulfatides (SHexCer) and phosphatidylethanolamine (PE) (each ~9%). Example images of control cells can be found in Figure S2 for PC-headgroup [C_5_H_15_PNO_4_]^+^ at *m*/*z* 184.07 and [FA 18:0-H]^−^ at *m*/*z* 283.26.

We identified 90 highly abundant lipids at the plasma membrane of human midbrain neurons, among which the major components are PCs (~33%), PSs (~11%), and Cers (~11%), followed by SHexCer and PE (each ~9%) (Figure 1). Mammalian neurons and brain tissue have been found with a high abundance of PCs and STs, followed by PEs, PSs, and Cers, according to the previous literature [4,75,76,77,78,79,80]; however, detailed information on native plasma membrane lipids of human neurons has been scarcely addressed. The midbrain region of the human and mouse brain was reported to contain PCs, PSs, PEs, and Cers [80,81]. In addition, we previously discovered Cers, PSs, PCs, and DGs as major lipid components of the plasma membrane of human one-week-old differentiating NPCs [82]. The results of these studies are aligned with the data of our current study in several aspects. The differences between our current data and our previous study are likely due to the different cell stages of human neuronal cells [4,75,76,77,78,79,80,81,82].

PCs are known to be amongst the most common lipids in neurons. Certain PC species detected here have previously been identified as highly abundant in neurons, such as PC 34:1, PC 36:1, PC 34:0, and PC 32:0, and their salt adducted compounds. These have been known to associate with important neuronal functions such as dopamine transport [82,83,84,85,86]. In addition, PSs were amongst the second most common lipids found in this study, and they are known to be prominent in neurons, and appear to increase during neuronal maturation [75]. They regulate mechanisms such as the prevention of neuronal apoptosis, maintenance of the cell membrane, storage of vital FAs, cell signaling, neurotransmission, and synapse formation. PSs also affect the progression of the midbrain disorder Parkinson’s disease, where certain PS species increase disease progression, whilst others can alleviate it. The varying effect of PSs on the midbrain could possibly relate to a strong PS-dependent cellular biology of neurons, thus explaining the high amount observed here [75,87,88,89,90,91].

Cers were found to be more abundant (~11%) than expected (~1% of whole cell lipid content) [76,79]; however, our previous study on differentiating NPCs also found Cers abundant (~24%) [82]. The higher amount of Cers detected in our studies compared to others could possibly be due to the differences in methodologies, analysis of the plasma membrane specifically, or cell types. Here, we employed ToF-SIMS to image intact plasma membrane lipids, while mass spectrometry bulk analysis of isolated cell membranes was used in other studies [75,76,77,79]. Cers are considered to regulate cell survival and cell death as well as to affect neuronal signaling, promotion of neurites and axons, metabolism, neuronal extracellular interactions, and synaptic transmission. Cers have also been observed to increase in midbrain dopaminergic neuronal maturation, which could explain the higher Cer content in our study [76,82,92]. Decreased levels of Cers and certain Cer species have been associated with Parkinson’s disease [92,93].

Another major lipid component is PEs, which is supported by other studies [4,77,79]. In the mouse midbrain, PEs appeared to be the second most common lipid among all phospholipids [86]. PEs are needed in FA storage, cell membrane curvature, ferroptosis indicator for phagocytosis, synaptic signaling, prevention of oxidative stress, and support in protein folding. The synthesis of PE, PC, and PS shares pathways that could, thus. relate to the high amount of these three lipids [15,86,94,95,96]. In Parkinson’s disease, ethanolamine was shown to protect dopaminergic neurons of *C. elegans* from alpha-synuclein, a pathological hallmark of the disease [94,95]. PE was also reported to decrease in the midbrain, especially in the substantia nigra pars compacta, of early Parkinson’s patients.

Finally, SHexCers were found as an abundant component, which is well correlated with the high abundance of Cers, since SHexCer synthesis is reliant on Cer synthesis [97,98]. However, it is difficult to relate our results to others because of the variation in the reported values. This variation is possibly due to the differences in the used cell type, organism, or analytical method, for example, in tissue isolation method tends to produce a high value because of a high amount of SHexCers in the myelin in the tissues [4,77,79,99,100]. SHexCers are important for immune regulation, extracellular interactions, membrane proteins, and maintenance of the action potential in the brain. SHexCers have also been associated with Parkinson’s disease [97,98]. Overall, the abundance of the above-discussed lipids could be related to the biology of mature midbrain neurons.

### 3.2. Effects of Lipid Head Groups on Neuronal Membrane Lipid Turnover—Ethanolamine Versus Choline

We further examined the lipid turnover of the plasma membrane in the midbrain neurons to identify how cells incorporate different precursors according to their specific biological demand and condition. In this section, isotopically labeled lipid headgroups, ^13^C_2_-ethanolamine or ^15^N-choline, were incubated in the cell cultures for 4 days before being removed from the cell medium for another 12 h. The change in lipid abundance in the isotopically incubated cells was then assessed by comparing the abundance of the respective lipids in control cells.

**Figure 2:**
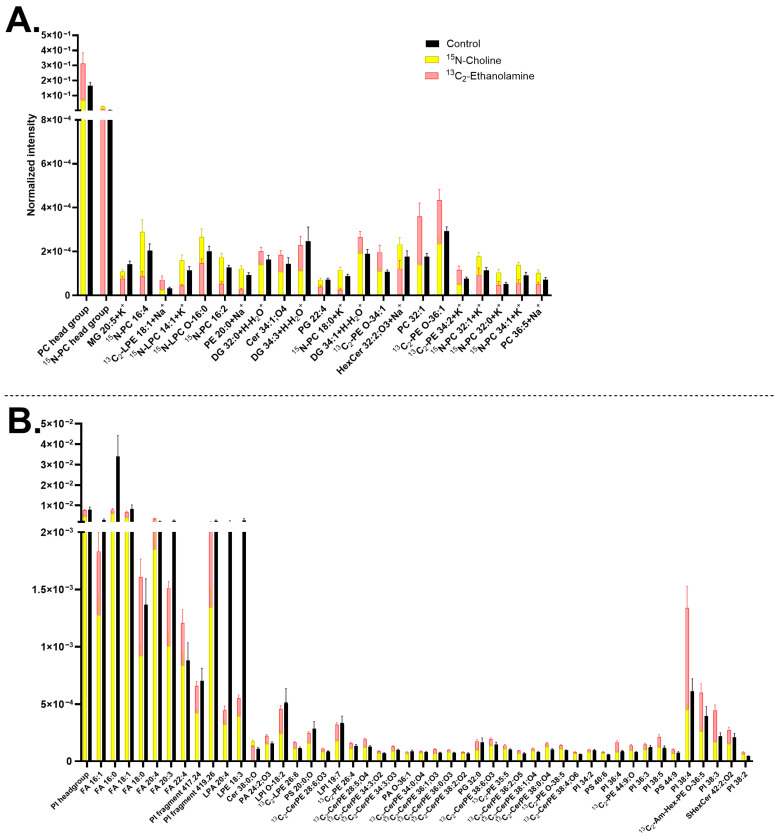
Major lipids with significantly different turnover in human midbrain neurons following ^13^C_2_-ethanolamine or ^15^N-choline incubation. (**A**) Lipids detected in positive mode and (**B**) lipids detected in negative mode. The ^13^C_2_-ethanolamine (pink) and ^15^N-choline (yellow) bars are superimposed, and the control (black) is interleaved. Lipids are arranged in each chart from left to right in ascending mass/charge (*m*/*z*). Error bars represent standard error of mean (SEM). The data of ^13^C_2_-ethanolamine and ^15^N-choline are significantly different from each other by a multiple Mann–Whitney test with a *p*-value of ≤0.05. Cells treated with either ^15^N-choline or ^13^C_2_-ethanolamine each had two biological replicates with seven to nine measurements per ion mode. The control had five biological replicates with 20 measurements per ion mode. 1-(1Z-alkenyl),2-acylglycerophosphoethanolamine glycan (Am-Hex-PE), ceramide (Cer), ceramide phosphoethanolamine (CerPE), diacylglycerol (DG), fatty acid (FA), hexosylceramide (HexCer), lysophosphatidic acid (LPA), lysohosphatidylcholine (LPC), lysophosphatidylethanolamine (LPE), lysophosphatidylinositol (LPI), monoacylglycerol (MG), phosphatidic acid (PA), phosphatidylcholine (PC), phosphatidylethanolamine (PE), phosphatidylglycerol (PG), phosphatidylinositol (PI), phosphatidylserine (PS), and sulfatide (SHexCer). Lipids with no annotated ionization were detected as [M+H]^+^ in (**A**) and [M–H]^−^ in (**B**).

The lipids contributing to the significant differences between the treated samples and the control are shown in Figure 2. Example ion images of several representative lipids are presented in Figure S3. The effects of ^15^N-choline incubation were mainly observed in positive mode (Figure 2A). Neurons incubated with ^15^N-choline incorporated the isotopic headgroup primarily into lysophosphatidylcholines (LPCs) and PCs, resulting in a decrease in the non-isotopic PC-headgroup species [C_5_H_15_PNO_4_]^+^ at *m*/*z* 184.07 (Figure S3A,C,D) and an increase in intensity of 15N-PC headgroup at *m*/*z* 185.07 (Figure 2A and Figure S3E,G,H). Other lipids, although having no choline headgroup in the structures, such as [PE 20:0+Na]^+^ at *m*/*z* 547.32, hexosylceramide (HexCer) [HexCer 32:2;O_3_+Na]^+^ at *m*/*z* 708.5, and [Cer 38:0;O-H]^−^ at *m*/*z* 578.59, also increased their turnover. In contrast, a few lipids containing no PC-headgroup were found decreased in their abundance, particularly [Cer 34:1;O_4_+H]^+^ at *m*/*z* 570.5 (Figure 2A), diacylglycerol (DG) [DG 34:3+H-H_2_O]^+^ at *m*/*z* 573.49, [FA 16:0-H]^−^ at *m*/*z* 255.23, [FA 18:0-H]^−^ at *m*/*z* 283.26, [PS 20:0;O-H]^−^ at *m*/*z* 582.3, and several phosphatidylinositols (PIs) and lysophosphatidylinositols (LPIs) (Figure 2B).

Midbrain neurons incubated with ^13^C_2_-ethanolamine appeared to have a higher number of lipids with increasing turnover compared to ^15^N-choline-incubated cells. The ^13^C_2_-ethanolamine headgroup was predominantly incorporated into ceramide phosphoethanolamines (CerPEs), followed by PEs, lysophosphatidylethanolamines (LPEs, Figure S3J,L), and PSs, all of which were mainly observed in the negative mode (Figure 2B).

Several other lipids containing no ethanolamine headgroup had an increased abundance such as PC-headgroup [C_5_H_15_PNO_4_]^+^ at *m*/*z* 184.07, [PC 32:1+H]^+^ at *m*/*z* 732.55 (Figure 2A), phosphatidic acid (PA) [PA 24:2;O_3_-H]^−^ at *m*/*z* 579.29, [SHexCer 42:2;O_2_-H]^−^ at *m*/*z* 888.62, and a few PI species (Figure 2B). [PS 40:6-H]^−^ at *m*/*z* 834.53 (Figure 2B) further increased its abundance following both ^13^C_2_-ethanolamine and ^15^N-choline treatment. In contrast, two lipids were found to decrease in turnover following ^13^C_2_-ethanolamine incubation, particularly a salt adduct of [HexCer 32:2;O_3_+Na]^+^ at *m*/*z* 708.5 and phosphatidylglycerol (PG) [PG 22:4+H]^+^ at *m*/*z* 575.3 (Figure 2A). In addition, both the isotopic incubations exhibited an opposite trend regarding the change in the abundance of [^13^C_2_-LPE 26:6-H]^−^ at *m*/*z* 582.34 (Figure 2B and Figure S3G–I), whereas they showed a similar decreasing trend in other lipids, such as monoacylglycerol (MG) [MG 20:5+K]^+^ at *m*/*z* 415.22 (Figure 2A), [FA 16:0-H]^−^ at *m*/*z* 255.23, lysophosphatidic acid (LPA) [LPA 20:4-H]^−^ at *m*/*z* 457.24, and [LPE 18:3-H]^−^ at *m*/*z* 474.26 (Figure 2B).

Comparing the results with high abundant lipids shown in Figure 1, several lipids were observed to overlap with the results of Figure 2, such as salt adducted PCs [^15^N-PC 32:0+K]^+^ at *m*/*z* 773.53, [^15^N-PC 32:1+K]^+^ at *m*/*z* 771.51, [^15^N-PC 34:1+K]^+^ at *m*/*z* 799.54 (Figure 2A), [^13^C_2_-PE O-38:5-H]^−^ at *m*/*z* 752.54, [PI 38:3-H]^−^ at *m*/*z* 887.57, [PI 38:4-H]^−^ at *m*/*z* 885.55, [PS 40:6-H]^−^ at *m*/*z* 834.53, and [SHex Cer 42:2;O_2_-H]^−^ at *m*/*z* 888.62 (Figure 2B).

PCs and PEs are among the major constituents of the neuronal plasma membrane. The difference in their lipid headgroup structures results in distinct molecular shapes. PCs containing a choline headgroup adopt a cylindrical shape, whereas PEs with an ethanolamine headgroup possess a conical shape [101,102]. These structural differences influence membrane properties, particularly fluidity and the dynamic formation of membrane curvature, which are critical for various neuronal processes, including exocytosis during neurotransmission [16,101,102]. Here, we investigated how these lipid headgroups contribute to the dynamic turnover of lipids within the neuronal plasma membrane. It was observed that the ^15^N-choline-incubated cells exhibit a high amount of turnover in many PCs, as expected [4]. However, ^15^N-choline appeared to incorporate into a smaller number of lipids and to a lower extent compared to ^13^C_2_-ethanolamine (Figure 2), which is contradictory to the higher abundance of PCs in neuronal plasma membrane (Figure 1). This may indicate a longer lifetime and, thus, slower turnover of choline-containing lipids compared to the ethanolamine-containing ones. Such an observation was also reported in rat brains and other cell types where PEs had a higher turnover than PCs [103,104]. Alternatively, the incubation with ^13^C_2_-ethanolamine might exert an overstimulating effect on the synthesis of ethanolamine-containing lipids.

Upon the cell incubation with ^13^C_2_-ethanolamine, many CerPEs were found to increase in abundance. This is a noteworthy result since CerPEs are thought to be less common than sphingomyelins (SMs) in mammalian cells [61,105]. SMs have a choline headgroup, but in our study, SMs were not found to increase in turnover upon ^15^N-choline incubation. Instead, ^13^C-ethanolamine incubation appeared to promote the synthesis and turnover of CerPEs, which contain ethanolamine headgroups in their structures. In addition, our findings are supported by several studies where CerPEs were detected in rat brain tissues [106], in rat brain microsomes and synaptic membrane [107], human NPCs [61], and in human and mouse brains using mass spectrometry imaging [106,108]. They were shown to have different properties and functions than SMs, and to be related to brain aging and Parkinson’s disease [106,109,110]. The total CerPE abundance in the plasma membrane is likely not high enough to be observed as common membrane lipids, since they were not detected within the 90 most abundant lipids. Our data illustrate the possibility of using specific isotopic precursor enrichment and ToF-SIMS imaging to explore the uncharted lipid organization and turnover aspects in neuronal plasma membrane.

### 3.3. Effects of the Unsaturation Level on Neuronal Membrane Lipid Turnover—Linolenic Acid Versus Linoleic Acid

The effects of the lipid precursor number and position of double bonds on neuronal membrane lipid turnover were examined by comparing the cell incubation with ^13^C-FA 18:3 omega-3 (^13^C-linolenic acid) and ^13^C-FA 18:2 omega-6 (^13^C-linoleic acid).

**Figure 3:**
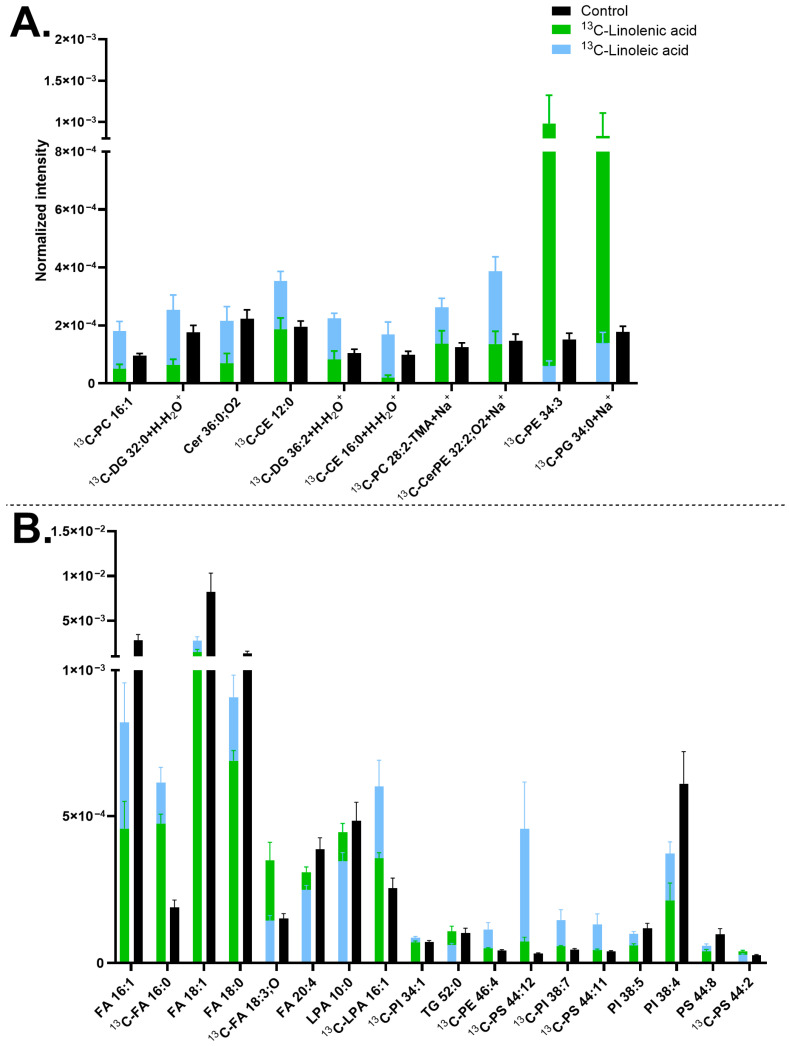
Lipid abundancy in mature human midbrain neurons upon ^13^C-linolenic acid and ^13^C-linoleic acid incubation. (**A**) Lipids detected in positive mode and (**B**) lipids detected in negative mode. Error bars represent standard error of mean (SEM). The ^13^C-linolenic acid (green) and ^13^C-linoleic acid (blue) bars are superimposed, and the control (black) is interleaved. All bars of ^13^C-linolenic acid and ^13^C-linoleic acid are significantly different from each other by a multiple Mann–Whitney test with a *p*-value of ≤0.05. Lipids are arranged from left to right in ascending mass per charge (*m*/*z*). Cells treated with either ^13^C-linolenic acid or with ^13^C-linoleic acid each had two biological replicates with seven to eight measurements per mode. The control had five biological replicates with 20 measurements in total per mode. Cholesterol ester (CE), ceramide (Cer), ceramide phosphoethanolamine (CerPE), diacylglycerol (DG), fatty acid (FA), lysophosphatidic acid (LPA), phosphatidylcholine (PC), phosphatidylethanolamine (PE), phosphatidylglycerol (PG), phosphatidylinositol (PI), phosphatidylserine (PS), and triglyceride (TG). Lipids with no annotated ionization were detected as [M+H^+^] in (**A**) and [M-H^−^] in (**B**).

The two ^13^C-FAs exhibited different trends regarding the effects on the lipid turnover. Particularly, ^13^C-linoleic acid was incorporated into many lipids, resulting in their higher abundance, such as DGs, PCs, and PSs, whereas ^13^C-linolenic acid maintained the abundance of most of these lipids similar to the control (Figure 3). ^13^C-linolenic acid increased the turnover of fewer lipids than its counterpart, including [^13^C-PE 34:3+H]^+^ at *m*/*z* 715.51, [^13^C-PG 34:0+Na]^+^ at 774.53 (Figure 3A), [^13^C-FA 18:3;O-H]^−^ at *m*/*z* 294.21, and [^13^C-PS 44:2-H]^−^ at *m*/*z* 899.65 (Figure 3B). Nevertheless, both ^13^C-FA incubations were found to have the same effects on several lipid compounds. For example, the turnover of [^13^C-FA 16:0-H]^−^ at 256.23, [^13^C-LPA 16:1-H]^−^ at *m*/*z* 408.22, and [^13^C-PS 44:12-H]^−^ at *m*/*z* 879.5 increased, whereas FAs, [PI 38:4-H]^−^ at *m*/*z* 885.55, and [PS 44:8-H]^−^ at *m*/*z* 886.56 decreased by both isotopic incubations (Figure 3B). The increased abundance of [^13^C-FA 18:3;O-H]^−^ at *m*/*z* 294.21 in ^13^C-linolenic acid incubated neurons compared to that of the ^13^C-linoleic acid incubated ones is shown in Figure S4A–C, whilst a higher abundance of [^13^C-PS 44:12-H]^−^ at *m*/*z* 879.5 in ^13^C-linoleic acid incubated cells is demonstrated in Figure S4D–F.

Linolenic acid (one of the three main types of omega-3 FAs) and linoleic acid (a type of omega-6 FAs) are essential FAs for brain function and development [111,112]. Omega-3 improves cell membrane fluidity and neurotransmission to promote cognition and protection against neurodegeneration [113,114], as well as preventing neural apoptosis by reducing the production of reactive oxygen species (ROS). On the other hand, while omega-6 is critical for brain development, an excess of it could induce brain inflammation and neurodegenerative diseases by promoting ROS accumulation [111]. Many studies have focused on the balance of omega FAs, where a balance of 3:1 of omega-6 to omega-3 has been linked to neurodevelopment and brain function, whilst a large imbalance appeared to cause negative effects [115,116]. Therefore, a balance between omega-3 and omega-6 is essential to health, neuronal function, cognition, learning, and memory [117]. The two FAs have quite similar molecular structures (C_18_ carbon chain), except for the number and position of the double bonds. We explored the effect of ^13^C-linolenic acid and ^13^C-linoleic acid on the neuronal membrane lipids and found that the incorporation of ^13^C-linoleic acid occurred in more lipids than ^13^C-linolenic acid. Both incubations decreased several non-isotopic FAs, such as [FA 16:1-H]^−^, [FA 18:1-H]^−^, and [FA 18:0-H]^−^. This could be due to a high amount of the isotopic precursors leading to their preferred incorporation into the cells.

Our results of a higher incorporation of ^13^C-linoleic acid than ^13^C-linolenic acid differ from previous studies using ToF-SIMS on the plasma membrane of PC12 cells [41,118], where a higher incorporation of ^13^C-linolenic acid was found. However, other studies on neural tissues and cells have reported similar results to our study [119,120,121,122,123,124,125]. Omega-6 intake was shown to play a role in inducing dopaminergic neurogenesis in the midbrain, indicating the relevance of our results for mature midbrain neurons [126]. ^13^C-linoleic acid incubated midbrain neurons had a high incorporation into DGs, PCs, and PSs. Prominent incorporation and reliance of omega-6 FAs, including linoleic acid in DGs, PCs, and PSs, has been previously observed in brain cells and other cell types [127,128,129,130,131,132,133]. In neurons, DGs in the plasma membrane have been shown to be important for neuronal synaptic formation and function [134]. In addition, omega-6 FAs have previously been found to incorporate into many phospholipids, which supports our observation. On the other hand, omega-3 FAs are more common in PEs or PSs [122,124,131,135,136], but we did not observe such a trend in these compounds here. Omega-3 FAs have been associated with differentiation [137], which could explain the lower incorporation of ^13^C-linolenic acid in mature neurons.

The two omega FAs share a lipid bioconversion pathway of elongation and unsaturation; however, our data indicate a distinctly molecular difference in membrane lipid turnover induced by these two FAs, which is consistent with previous observations [117,138]. This could be the key factor contributing to the difference in their neurological effects.

### 3.4. Effects of Carbon Chain Saturation of Lipid Precursor on Neuronal Membrane Lipid Turnover—Linoleic Acid and Linolenic Acid Versus Stearic Acid

We next examined the neuronal membrane lipid turnover, comparing cell incubations with ^13^C^−^linoleic acid, ^13^C^−^linolenic acid, and ^13^C-stearic acid. These FAs have the same carbon chain (C_18_) but differ in their degree of unsaturation. A three-component PCA was initially performed to compare the three sample groups, which showed some overlap. Therefore, a two-component PCA was used to better highlight the similarities and differences among the samples.

**Figure 4:**
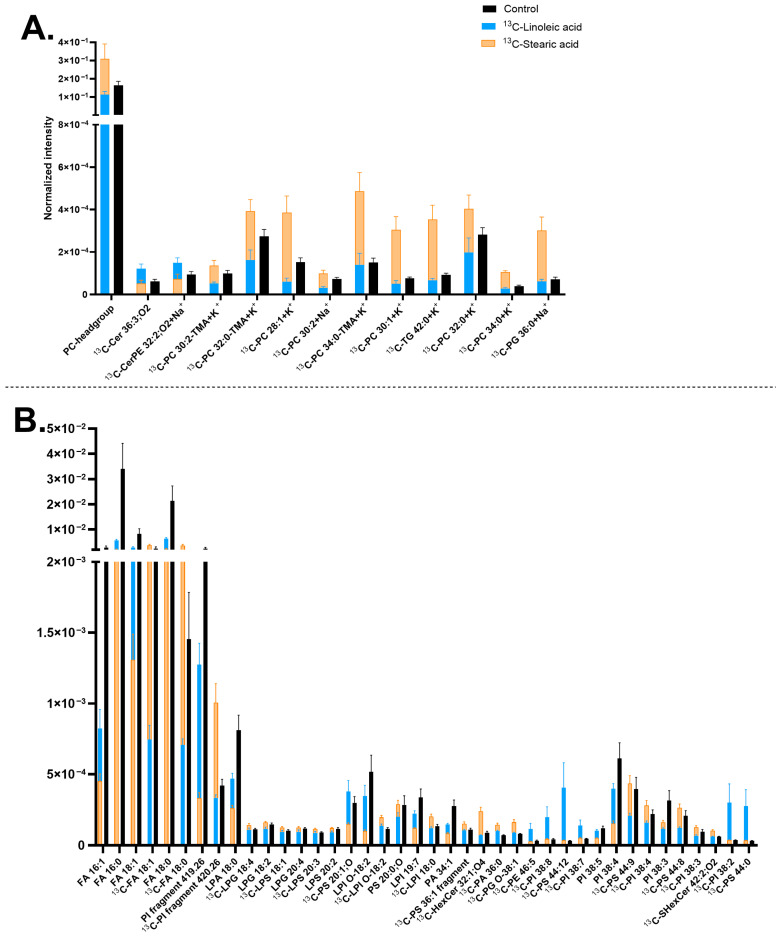
Lipid abundancy in mature human midbrain neurons upon ^13^C-linoleic acid or ^13^C-stearic acid incubation. (**A**) Lipids detected in positive mode; (**B**) Lipids detected in negative mode. Error bars represent standard error of mean (SEM). The ^13^C-stearic acid (orange) and ^13^C-linoleic acid (blue) bars are superimposed, and the control (black) is interleaved. All bars of ^13^C-stearic acid and ^13^C-linoleic acid are significantly different from each other by a multiple Mann–Whitney test with a *p*-value of ≤0.05. Lipids are arranged (**A**,**B**) from left to right in ascending mass per charge (*m*/*z*). ^13^C-linoleoic acid incubated samples had two biological replicates with four measurements per ion mode. ^13^C-stearic acid incubated samples had two biological replicates with seven to eight measurements per ion mode, and the control had five biological replicates with 20 measurements per ion mode. Ceramide (Cer), ceramide phosphoethanolamine (CerPE), fatty acid (FA), hexosylceramide (HexCer), lysophosphatidic acid (LPA), lysophosphatidylglycerol (LPG), lysophosphatidylinositol (LPI), lysophosphatidylserine (LPS), phosphatidic acid (PA), phosphatidylcholine (PC), phosphatidylethanolamine (PE), phosphatidylglycerol (PG), phosphatidylinositol (PI), phosphatidylserine (PS), sulfatide (SHexCer), and triglyceride (TG). Lipids with no annotated ionization were detected as [M+H]^+^ in (**A**) and [M-H]^−^ in (**B**).

It was observed that ^13^C-stearic acid is generally incorporated into a larger number of neuronal membrane lipids than the ^13^C-linoleic acid precursor (Figure 4). Particularly, all the ^13^C-PCs (correspondingly the PC headgroup at *m*/*z* 184.07) and [^13^C-PI fragment] at *m*/*z* 420.26 increased their concentrations in the ^13^C-stearic acid incubated samples. Several ^13^C-LPIs also increased, such as [^13^C-LPI O-18:2-H]^−^ at *m*/*z* 582.31 and [^13^C-LPI 18:0-H]^−^ at *m*/*z* 600.32, which is likely corresponding to the elevated amount of [^13^C-PI fragment] at *m*/*z* 420.26. In addition, the ^13^C-stearic acid precursor [^13^C-FA 18:0-H]^−^ at *m*/*z* 284.26 was observed. An example of the high PC incorporation is shown in Figure S5 via the PC-headgroup [C_5_H_15_PNO_4_]^+^ at *m*/*z* 184.07 and [^13^C-PC 30:1+K]^+^ at *m*/*z* 743.48 (Figure 4A and Figure S5D–F). The incorporation of [^13^C-PI fragment] at *m*/*z* 420.26 is shown in Figure S5G–I.

^13^C-linoleic acid is incorporated primarily into PIs and PSs, mostly with high double bonds, such as [^13^C-PI 38:8-H]^−^ at *m*/*z* 878.49, [^13^C-PI 38:7-H]^−^ at *m*/*z* 880.5, [^13^C-PI 38:2-H]^−^ at *m*/*z* 890.58, [^13^C-PS 44:12-H]^−^ at *m*/*z* 879.5, except for [^13^C-PS 44:0-H]^−^ at *m*/*z* 903.69 (Figure 4B). The ion images of [^13^C-PI 38:7-H]^−^ at *m*/*z* 880.5 are shown in Figure S5J–L. In addition, the two isotopic precursors showed an opposite effect on [^13^C-FA 18:0-H]^−^ at *m*/*z* 284.26 (^13^C-stearic acid) (Figure 4B) and several ^13^C-PCs (Figure 4A), which have higher abundance with ^13^C-stearic acid but lower abundance with ^13^C-linoleic acid. Finally, both precursors exerted similar negative effects on the turnover of a few FAs and PIs, particularly non-isotopic lipid compounds [FA 16:0-H]^−^ at *m*/*z* 255.23, [FA 16:1-H]^−^ at *m*/*z* 253.22, [FA 18:0-H]^−^ at *m*/*z* 283.26, [PI 38:4-H]^−^ at *m*/*z* 885.55, and [PI 38:3-H]^−^ at 887.57 (Figure 2B).

Compared with the results in Figure 3, similar trends were observed for several lipids. For example, [^13^C-CerPE 32:2;O_2_+Na]^+^ at *m*/*z* 654.46, [^13^C-PI 38:7-H]^−^ at *m*/*z* 880.5, and [^13^C-PS 44:12-H]^−^ at *m*/*z* 879.5 showed increased abundance whereas [FA 18:0-H]^−^ at *m*/*z* 283.26, [FA 18:1-H]^−^ at *m*/*z* 281.25, [PI 38:4-H]^−^ at *m*/*z* 885.55, and [PI 38:5-H]^−^ at *m*/*z* 883.53 showed decreased abundance. Additionally, comparison with Figure 1 reveals that many of significantly turned-over lipids identified in Figure 4 are major components of neuronal membrane lipids, including PCs (Figure 4A), FAs ([FA 16:0-H]^−^ at *m*/*z* 255.23, [FA 18:0-H]^−^ at *m*/*z* 283.26, and [FA 18:1-H]^−^ at *m*/*z* 281.25), LPIs, and PIs (Figure 4B).

**Figure 5:**
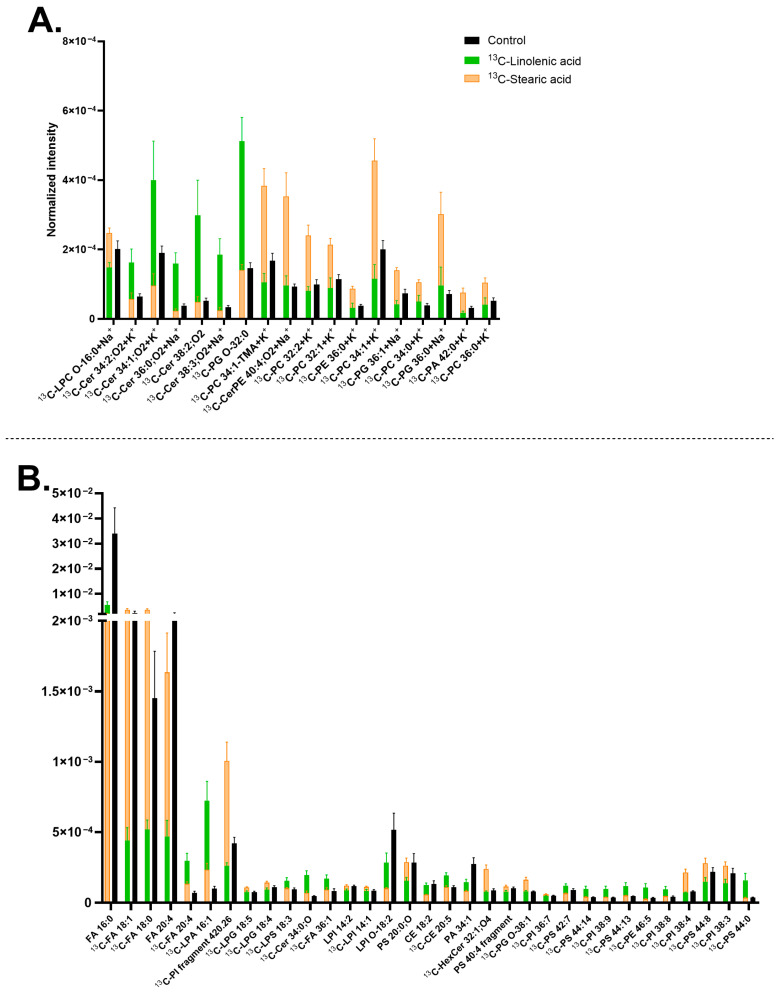
Lipids abundance in mature human midbrain neurons upon ^13^C-linolenic acid or ^13^C-stearic acid incubation. (**A**) Lipids detected in positive mode; (**B**) Lipids detected in negative mode. Error bars represent standard error of mean (SEM). The ^13^C-stearic acid (orange) and ^13^C-linolenic acid (green) bars are superimposed, and the control (black) is interleaved. All bars of ^13^C-stearic acid and ^13^C-linolenic acid are significantly different from each other by a multiple Mann–Whitney test with a *p*-value of ≤0.05. Lipids are arranged (**A**,**B**) from left to right in ascending mass per charge (*m*/*z*). ^13^C-linolenic acid and ^13^C-stearic acid incubated samples had two biological replicates with seven to eight measurements per ion mode. The control had five biological replicates with 20 measurements per ion mode. Cholesterol ester (CE), ceramide (Cer), ceramide phosphoethanolamine (CerPE), fatty acid (FA), hexosylceramide (HexCer), lysophosphatidic acid (LPA), lysophosphatidylglycerol (LPG), lysophosphatidylinositol (LPI), lysophosphatidylserine (LPS), phosphatidic acid (PA), phosphatidylcholine (PC), phosphatidylethanolamine (PE), phosphatidylglycerol (PG), phosphatidylinositol (PI), and phosphatidylserine (PS). Lipids with no annotated ionization were detected as [M+H]^+^ in (**A**) and [M–H]^−^ in (**B**).

Next, lipid turnover of midbrain neurons was compared between ^13^C-stearic acid and ^13^C-linolenic acid incubations. Upon ^13^C-stearic acid treatment, PCs and several PIs significantly increased their abundance compared to the control and ^13^C-linolenic samples, such as [^13^C-PC 34:1+K]^+^ at *m*/*z* 799.54 (Figure 5A and Figure S6A–C) and ^13^C-PI fragment [C_21_H_40_O_6_P]^−^ at *m*/*z* 420.26 (Figure 5A and Figure S6D–F). The precursor also induced higher abundance of [^13^C-FA 18:1-H]^−^ at *m*/*z* 282.25 and [^13^C-FA 18:0-H]^−^ at *m*/*z* 284.26 (precursor itself) (Figure S6G–I). On the other hand, ^13^C-linolenic acid incubation exhibited an increased amount of Cers and some PSs, such as [^13^C-Cer 34:1;O_2_+K]^+^ at *m*/*z* 577.48 (Figure 5A and Figure S6J–L), and [^13^C-PS 44:13-H]^−^ at *m*/*z* 877.48 (Figure 5B and Figure S6M–O). Among these lipids, some contained a high double bond number, for example [^13^C-PS 44:13-H]^−^ at *m*/*z* 877.48 and [^13^C-PS 44:14-H]^−^ at *m*/*z* 875.47. In addition, both incubations increased the amount of [^13^C-FA 20:4-H]^−^ at *m*/*z* 304.23 and [^13^C-LPA 16:1-H]^−^ at *m*/*z* 408.22, with ^13^C-linolenic acid showing a greater extent. In contrast, they decreased the level of [FA 16:0-H]^−^ at *m*/*z* 255.23, [FA 20:4-H]^−^ at *m*/*z* 303.23, and [LPI O-18:2-H]^−^ at *m*/*z* 581.31 (Figure 5B).

In relation to Figure 3, which compares ^13^C-linolenic acid and ^13^C-linoleic acid, some lipids were common, such as [^13^C-LPA 16:1-H]^−^ at *m*/*z* 408.22, having higher abundance with ^13^C-linolenic acid (Figure 3B and Figure 5B). On the other hand, [^13^C-PI 38:4-H]^−^ at *m*/*z* 886.55 and [^13^C-PS 44:8-H]^−^ at *m*/*z* 887.56 showed higher abundance with ^13^C-stearic acid. In addition, comparing Figure 4 and Figure 5, similar lipids were observed, for example, [FA 16:0-H]^−^ at *m*/*z* 255.23 with lower abundance, whereas [^13^C-FA 18:1-H]^−^ at *m*/*z* 282.25, [^13^C-FA 18:0-H]^−^ at *m*/*z* 284.26, and PCs having higher abundance with ^13^C-stearic acid.

The difference in lipid turnover compared between ^13^C-linoleic acid, ^13^C-linolenic acid, and ^13^C-stearic acid incubation (Figure 4 and Figure 5) revealed that the largest number of lipids have the isotopic incorporation with ^13^C-stearic acid, particularly all the ^13^C-PCs, ^13^C-PI fragment 4.26 [C_21_H_40_O_6_P]^−^, and several ^13^C-LPIs. On the other hand, fewer lipids were found to turn over by ^13^C-linoleic acid, particularly PIs and PSs with high double bonds, such as [^13^C-PI 38:8-H]^−^ at *m*/*z* 878.49, [^13^C-PI 38:7-H]^−^ at *m*/*z* 880.5, [^13^C-PI 38:2-H]^−^ at *m*/*z* 890.58, and [^13^C-PS 44:12-H]^−^ at *m*/*z* 879.5. ^13^C-linolenic acid was also incorporated into a few lipids, primarily Cers and PSs, for example [^13^C-Cer 38:2;O_2_+H]^+^ at *m*/*z* 593.57, [^13^C-Cer 34:1,O_2_+K]^+^ at *m*/*z* 577.48, [^13^C-PS 42:7-H]^−^ at *m*/*z* 861.54, and [^13^C-PS 44:13-H]^−^ at *m*/*z* 877.48. Cells incubated with ^13^C-stearic acid increased the abundance of lighter lipids (C_28_-C_42_ with ^13^C-stearic acid, C_30_-C_46_ for ^13^C-linoleic and ^13^C-linolenic acids) with fewer double bonds (0–8 for ^13^C-stearic acid, 0–14 for ^13^C-linoleic and ^13^C-linolenic acid). This is expected due to their molecular structures, and unsaturated FAs tend to produce cellular lipids that are elongated and unsaturated [117,138]. PSs and PIs have been found to contain omega-3 FAs and omega-6 FAs in the brain [15,139,140,141,142]. A low omega-3 intake also resulted in a decrease in PS lipids in the brain [15,139,140]. These lipids are known to affect cellular signaling, synaptic membrane fusion, and regulation of glutamate receptors, which are located in midbrain neurons. The higher turnover of ^13^C-stearic acid is consistent with our previous report in human differentiating NPCs with ^13^C-stearic acid [82]. The lower plasma membrane lipid turnover of ^13^C-linoleic acid compared to ^13^C-stearic acid could be explained by other findings that over half the amount of linoleic acid entering the brain is used in energy production [120,143]. In addition, it was found that the in vivo turnover of omega FAs is considerably slow and may take up to several weeks [120,138].

The ^13^C-stearic acid incubated cells showed an increased abundance of [^13^C-FA 18:0-H]^−^ at *m*/*z* 284.26, indicating a high accumulation of the isotopic precursor after the incubation. Other studies have shown a similar trend of non-isotopic stearic acid and the lipids containing the stearic acid chain in neuronal cells and the brain [82,144,145,146,147,148,149]. The strong preference for stearic acid observed here is likely attributed to its high turnover and abundance in the brain, along with its frequent incorporation into PCs [82,141,148,149]. In addition, in our previous study on human NPCs, a high incorporation of ^13^C-stearic acid was shown to occur mainly into Cers and DGs, and a few PCs. Thus, the lipid turnover is different between the two cell stages with ^13^C-stearic acid, and this could be elaborated that Cers and DGs have a higher abundance during neuronal differentiation [82,150,151]. Our data show that stearic acid is a preferred precursor for PCs. In the mouse midbrain, PCs containing a FA 18:0 chain are amongst the common lipids, following PSs, PIs, and SHexCers [152], and they also incorporate ^13^C-stearic acid. The results suggest that ^13^C-stearic acid is one of the most efficiently incorporated lipid precursors in human neuronal cells.

### 3.5. Effect of Carbon Chain Length in Neuronal Membrane Lipid Turnover—Stearic Acid Versus Lauric Acid

**Figure 6:**
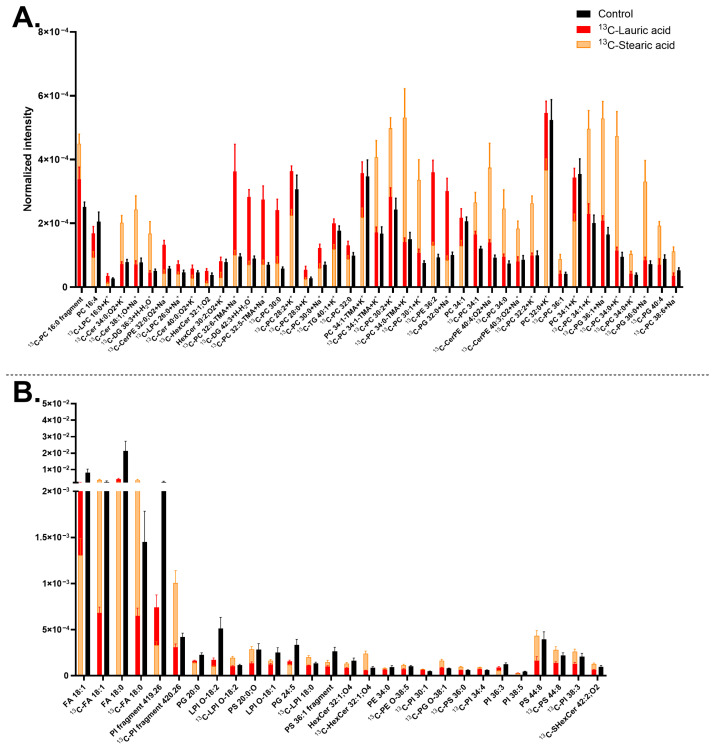
Lipid abundancy in mature human midbrain neurons upon ^13^C-lauric acid or ^13^C-stearic acid incubation. (**A**) Lipids detected in positive mode; (**B**) Lipids detected in negative mode. Error bars represent standard error of mean (SEM). The ^13^C-stearic acid (orange) and ^13^C-lauric acid (red) bars are superimposed, and the control (black) is interleaved. All bars of ^13^C-stearic acid and ^13^C-lauric acid are significantly different from each other by a multiple Mann–Whitney test with a *p*-value of ≤0.05. Lipids are arranged from left to right in ascending mass per charge (*m*/*z*). ^13^C-stearic acid incubated samples had two biological replicates with six to eight measurements per ion mode. ^13^C-lauric acid incubated samples had two biological replicates with five to eight measurements per mode, and the control had five biological replicates with 20 measurements per mode. Ceramide (Cer), ceramide phosphoethanolamine (CerPE), diacylglycerol (DG), fatty acid (FA), hexosylceramide (HexCer), lysophosphatidic acid (LPA), lysohosphatidylcholine (LPC), lysophosphatidylinositol (LPI), phosphatidylcholine (PC), phosphatidylethanolamine (PE), phosphatidylglycerol (PG), phosphatidylinositol (PI), phosphatidylserine (PS), sulfatide (SHexCer), and triglyceride (TG). Lipids with no ionization annotated were detected as [M+H]^+^ in (**A**) and [M–H]^−^ in (**B**).

In this section, we studied the role of carbon chain length on the membrane lipid turnover by comparing ^13^C-stearic acid (FA 18:0) and ^13^C-lauric acid incubations (FA 12:0), often referred to as medium- and short-chain FAs, respectively. Incubation with ^13^C-stearic acid resulted in the turnover of a larger number of lipids than ^13^C-lauric acid (Figure 6). Both precursors were incorporated into many PCs, PGs, and several Cers; however, ^13^C-lauric acid appears to be favored by lipids with shorter carbon chains (from C_28_ to C_32_), whereas ^13^C-stearic acid is preferred by compounds with longer carbon chains (from C_30_ to C_38_). Both were also incorporated mainly into lipids with a low unsaturation level (0 and 1 double bond in the carbon chain).

Midbrain neurons treated with ^13^C-stearic acid generally decreased the turnover of non-isotopic lipids, for example [PC 16:4+H]^+^ at *m*/*z* 502.26, [HexCer 30:2;O_2_+K]^+^ at *m*/*z* 680.45, [PC 34:1-TMA+K]^+^ at *m*/*z* 739.47, [PC 34:1+H]^+^ at *m*/*z* 760.59 (Figure 6A), [FA 18:1-H]^−^ at *m*/*z* 281.25, and [FA 18:0-H]^−^ at *m*/*z* 283.26 (Figure 6B), but increased the concentration of many isotopic counterparts of these compounds. In addition, ^13^C-stearic acid increased several Cers (e.g., [^13^C-Cer 38:1;O+Na]^+^ at *m*/*z* 601.57), CerPEs (e.g., [^13^C-CerPE 40:4;O_2_+Na]^+^ at *m*/*z* 762.56), and PIs (e.g., [^13^C-LPI O-18:2-H]^−^ at *m*/*z* 582.31, ^13^C-PI fragment 420.26 [C_21_H_40_O_6_P]^−^(Figure S7M–O), notably the PIs only showed increase trend with ^13^C-stearic acid. This indicates that PI turnover seems connected to only ^13^C-stearic acid, not ^13^C-lauric acid. Ion images of [LPC 16:0+K]^+^ at *m*/*z* 535.3, [^13^C-PS 36:0-H]^−^ at *m*/*z* 791.56 and several other lipids are shown in Figure S7. Finally, these detected lipids were within the highly abundant lipids in human neuronal plasma membrane shown in Figure 1, such as FAs, PCs, LPIs, Cers, HexCers, and PSs.

Both precursors were found to incorporate into many lipids, including PCs, PGs, and several Cers, most of them with a low number of double bonds (0 or 1 double bond). In addition, ^13^C-lauric acid was favored for lipids with shorter carbon chains than ^13^C-stearic acid (range of C_28_-C_32_ and C_30_-C_38_, respectively). This is expected due to the saturation state and length of the carbon chain of these precursors. The comparison between ^13^C-lauric acid and ^13^C-stearic acid has also been performed previously in our lab on human differentiating NPCs using ToF-SIMS with a (CO_2_)_6000_^+^ GCIB, which showed that ^13^C-lauric acid and ^13^C-stearic acid were incorporated mainly into Cers and DGs, but not PCs [82]. The shift in lipid turnover, with increased incorporation into PCs by ^13^C-lauric acid and ^13^C-stearic acid in this study, showed a similar trend compared to the results for ^13^C-stearic acid and ^13^C-linoleic acid. Lipid turnover consistently shifting from Cers and DGs to PCs may reflect the transition from NPCs to mature midbrain neurons. Overall, ^13^C-lauric acid induced fewer lipids to increase turnover than ^13^C-stearic acid, possibly because the brain favors longer-chain FAs. In addition, shorter-chain FAs are more likely to be used for energy metabolism compared to longer-chain FAs [75,82,153,154,155].

## 4. Conclusions

In this study, we aimed to characterize the molecular organization and dynamic turnover of the major plasma membrane lipids in human midbrain neurons to advance understanding of neuronal biology in relation to membrane lipids. Using ToF-SIMS imaging and the shotgun approach, we identified the predominant neuronal plasma membrane lipids and compared the molecular turnover patterns of the membrane lipids using different lipid precursors, generating a comprehensive dataset with new insights into the membrane composition of human midbrain neurons.

The lipid profile and turnover pattern, which are strikingly characterized by a high prevalence of PCs, PSs, Cers, and a preference for utilizing stearic acid as a precursor, could be directly linked to the biological state and functions of midbrain neurons. In addition, the lipid turnover alters drastically depending on the types of precursors, indicating a significant role of these exogenous molecules in mediating the molecular structure of neuronal membranes, and, hence, influencing neuronal functions. This may have a significant implication for future medical intervention. These findings provide a groundwork for research on lipid turnover and metabolism across neuronal developmental stages, different neuronal cell types, and under varying precursors or physiological conditions. Our work demonstrates the potential of ToF-SIMS imaging to investigate neuronal plasma membrane lipid abundance and turnover, elucidating the dynamic molecular structure of the neuronal plasma membrane and its link to neuronal functions.

## Data Availability

The original contributions presented in this study are included in the article/Supplementary Material. Further inquiries can be directed to the corresponding author.

## Supplementary Materials

The following supporting information can be downloaded at: https://www.mdpi.com/article/10.3390/biom15121650/s1, Figure S1: Immunocytochemistry images of neural progenitor cells (NPCs) and differentiated mature midbrain neurons; Figure S2: Example ToF-SIMS images of mature human midbrain neurons samples; Figure S3: Comparison of plasma membrane lipid turnover in mature human midbrain neurons incubated with 15N-choline and 13C2-ethanolamine headgroup precursors; Figure S4: Comparison of plasma membrane lipid turnover in mature human midbrain neurons incubated with 13C-linoleic acid and 13C-linolenic acid; Figure S5: Comparison of plasma membrane lipid turnover in mature human midbrain neurons incubated with 13C-linoleic acid and 13C-stearic acid; Figure S6: Comparison of plasma membrane lipid turnover in mature human midbrain neurons incubated with 13C-linolenic acid and 13C-stearic acid; Figure S7: Comparison of plasma membrane lipid turnover in mature human midbrain neurons incubated with 13C-lauric acid and 13C-stearic acid; Table S1: Summary of identified lipids in positive mode; Table S2: Summary of identified lipids in negative mode.
