# Supplementary material for: Plasma Membrane Lipid Composition and Turnover in Human Midbrain Neurons Investigated by Time-of-Flight Mass Spectrometry
Source: Biomolecules. 2025 Nov 24;15(12):1650. doi: 10.3390/biom15121650 (PMC12730232; doi:10.3390/biom15121650)
Supplement: Supplementary file 1 [file biomolecules-15-01650-s001.zip › biomolecules-3950177-supplementary.pdf]

Supplementary data

Plasma Membrane Lipid Composition and Turnover in Human Midbrain Neurons Investigated by Time-of-Flight Mass Spectrometry

Emmanuel Berlin[1], Alicia A. Lork[1], Carl Ernst[2], John S. Fletcher[1], and Nhu T.N. Phan[1*]

[1] Department of Chemistry and Molecular Biology, University of Gothenburg, 405 30 Gothenburg, Sweden. nhu.phan@chem.gu.se; emmanuel.berlin@gu.se; alicia.lork@googlemail.com; john.fletcher@chem.gu.se
[2] McGill University, Montreal Neurological Institute, *QC H3A 0G4* Montreal, Canada. carl.ernst@mcgill.ca
* Correspondence: nhu.phan@chem.gu.se

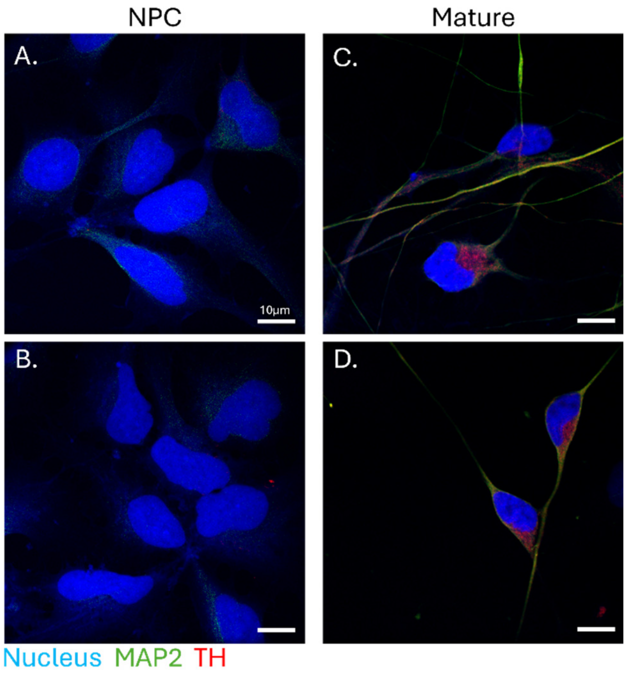

Figure S1. Immunocytochemistry images of neural progenitor cells (NPCs) and differentiated mature midbrain neurons. (**A** and **B**) NPCs and (**C** and **D**) mature midbrain neurons are stained for detection of nucleus (blue), microtubule-associated protein 2 (MAP2, green), and tyrosine hydroxylase (TH, red).

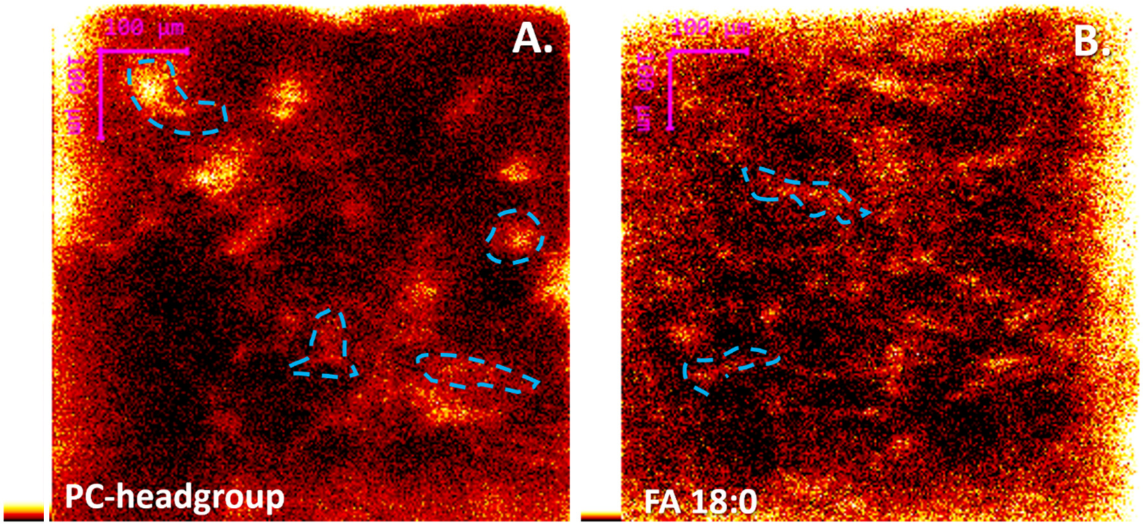

Figure S2. Example ToF-SIMS images of mature human midbrain neurons samples. The samples were imaged at 256 x 256 pixels showing signal intensities of either phosphatidylcholine (PC) headgroup $[C_5H_{15}PNO_4]^+$, m/z 184.07 (**A**) in positive mode or fatty acid (FA) $[FA\ 18{:}0\text{-}H]^-$ at m/z 283.26 (**B**) in negative mode. Blue dashed lines show examples of single midbrain neurons. Peak intensity scale on the left of each image is from the lowest intensity (black) to highest (white), and scale bars are 100 µm.

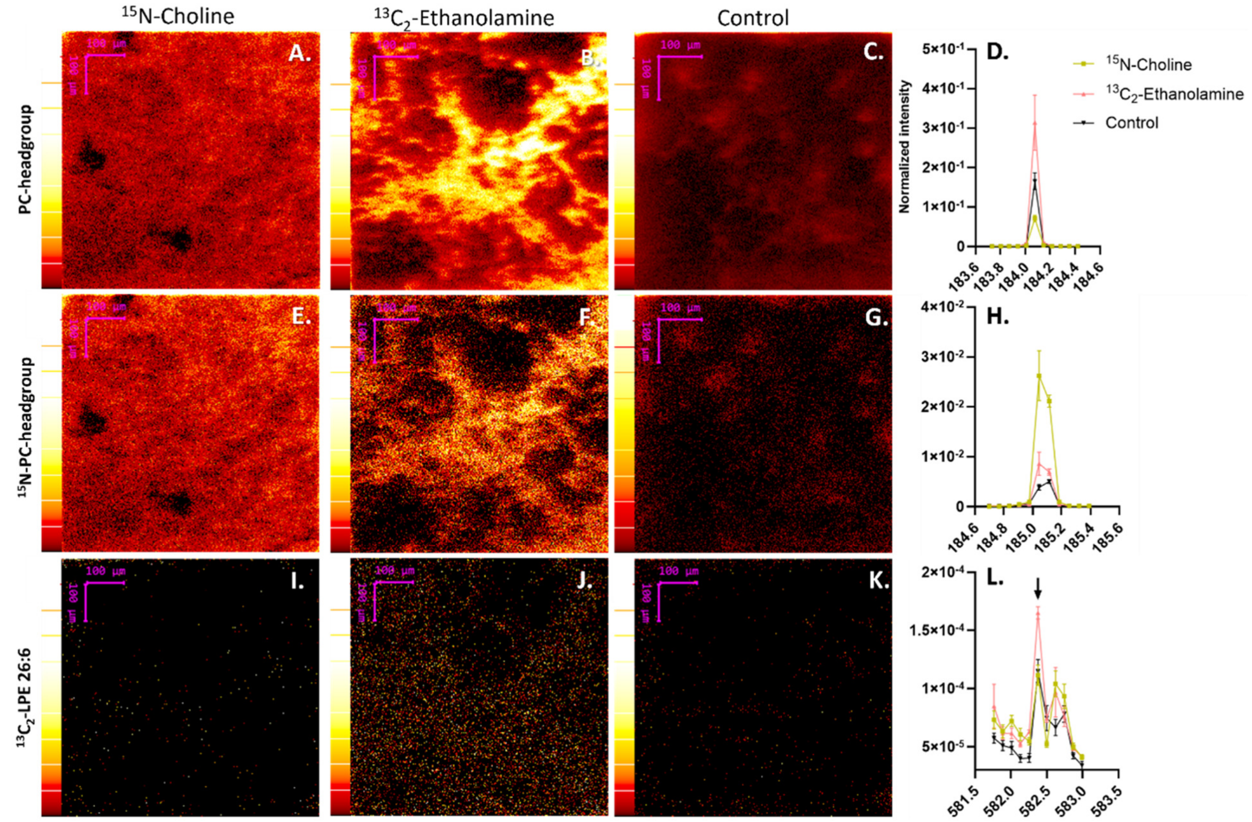

Figure S3. Comparison of plasma membrane lipid turnover in mature human midbrain neurons incubated with ^{15}N-choline and $^{13}C_2$-ethanolamine headgroup precursors. ToF-SIMS ion images at 256 x 256 pixels show the signal intensity of various lipids. (**A**-H) Positive mode; (**I**-**L**) negative mode; (**A**, **E**, and **I**) ^{15}N-choline treated midbrain neurons; (**B**, **F**, and **J**) ^{13}2C-ethanolamine treated midbrain neurons; (**C**, **G**, and **K**) control cells; (**A**-**D**) PC-headgroup at m/z 184.07; (**E**-**H**) ^{15}N-PC headgroup at m/z 185.04; (**I**-**L**) $^{13}C_2$-lysophosphatidylethanolamine (LPE) 26:6 at m/z 582.34. Peak intensity scale on the left of each image is from the lowest intensity (black) to highest (white), and scale bars are 100 µm. Fig **S3D**, **H**, and **L** compare the peak intensities of the imaged ions between the three treatment conditions. The peak intensity was binned and normalized to the total ion counts within the cell areas. Y-axis represents the normalized spectra, and the x-axis shows m/z. Error bars shown as standard error mean (SEM).

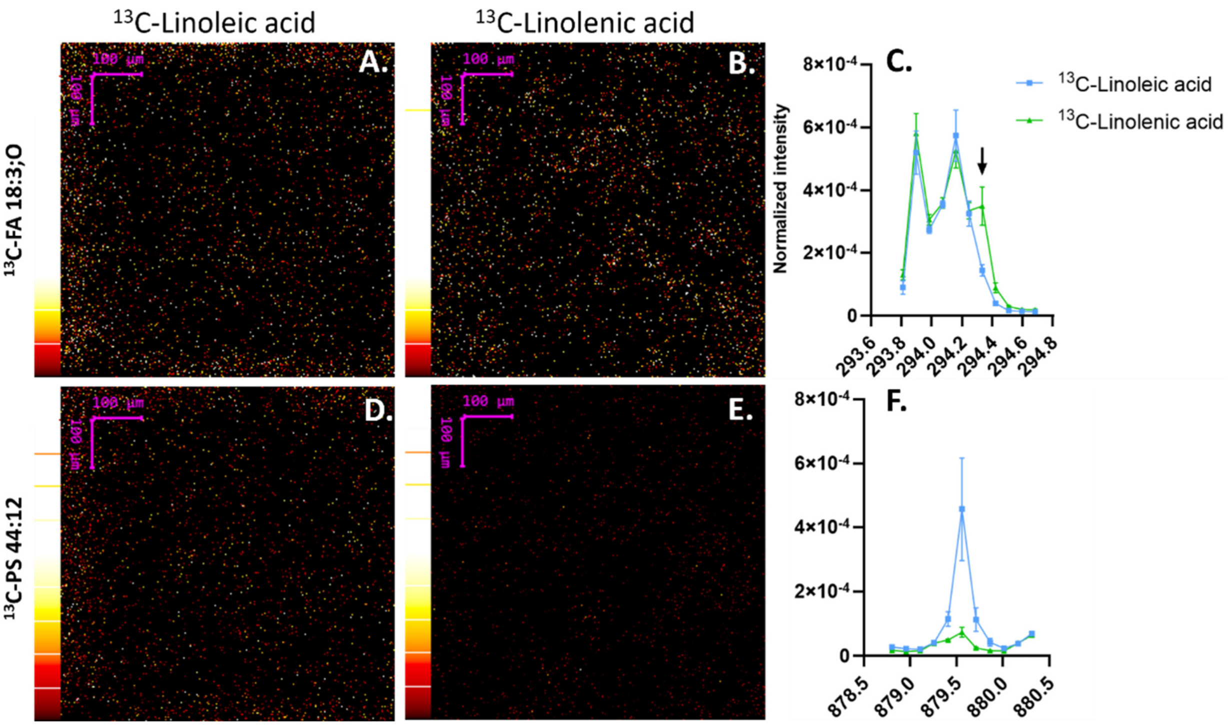

Figure S4. Comparison of plasma membrane lipid turnover in mature human midbrain neurons incubated with ^{13}C-linoleic acid and ^{13}C-linolenic acid. SIMS ion images at 256 x 256 pixels show the signal intensity of various lipids. (**A-D**) Negative mode, lipids ionized as $[M\text{-}H]^-$; (**A** and **D**) ^{13}C-linoleic acid treated midbrain neurons; (**B** and **E**) ^{13}C-linolenic acid treated midbrain neurons; (**A-C**) ^{13}C-fatty acid (FA) 18:3;O at m/z 294.21; (**D-F**) ^{13}C-phosphatidylserine (PS) 44:12 at m/z 879.5. Peak intensity scale on the left of each image is from the lowest intensity (black) to highest (white), and scale bars are 100 µm. Fig **S4C and F** compare the peak intensities of the imaged ions between the two treatment conditions. The peak intensity was binned and normalized to the total ion counts within the cell areas. Y-axis represents the normalized spectra, and the x-axis shows m/z. Error bars shown as standard error mean (SEM).

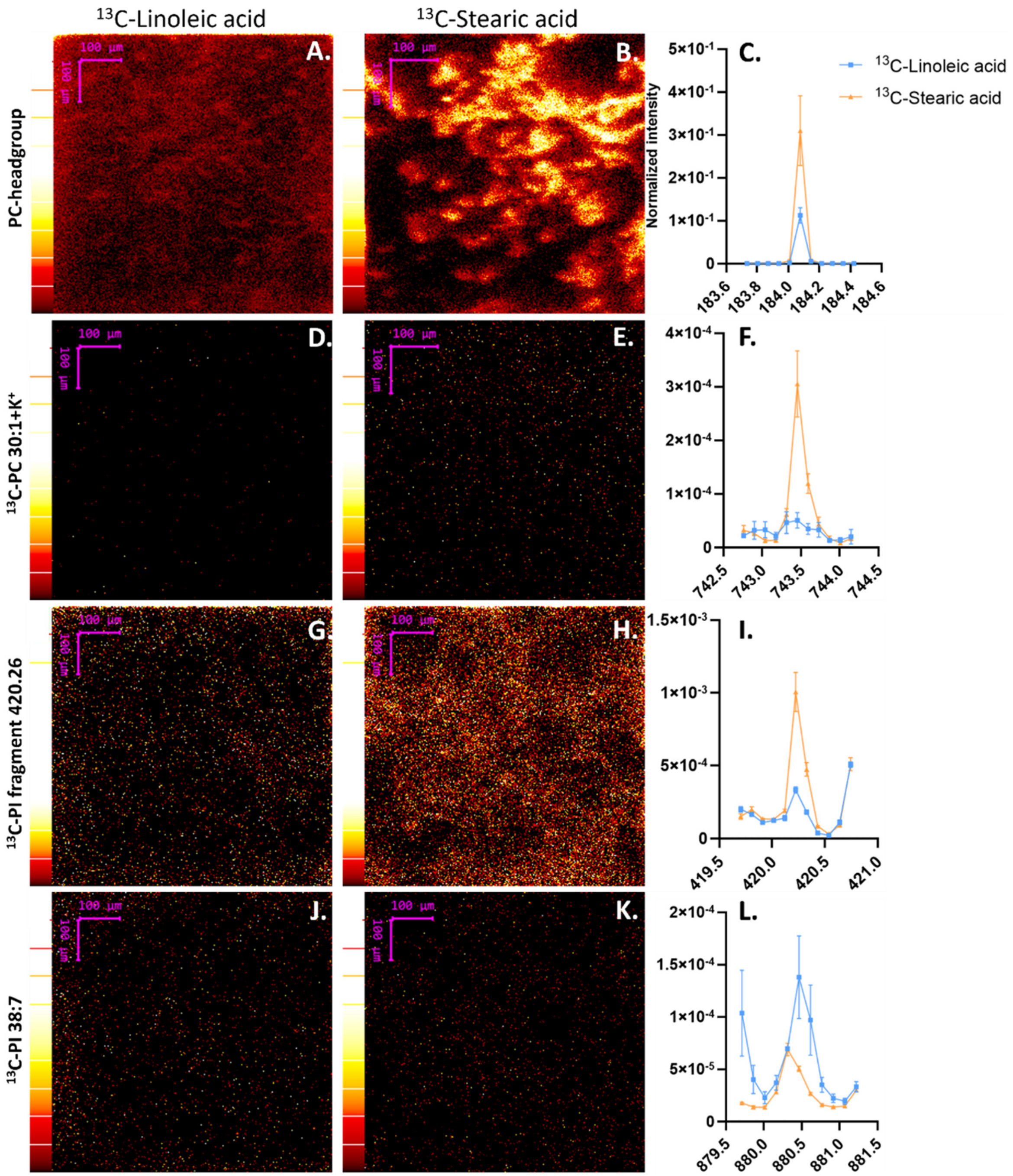

Figure S5. Comparison of plasma membrane lipid turnover in mature human midbrain neurons incubated with ^{13}C-linoleic acid and ^{13}C-stearic acid. SIMS ion images at 256 x 256 pixels show the signal intensity of various lipids. (**A**-**F**) Positive mode; (**E**-**L**) negative mode; (**A**,**D**, **G**, and **J**) ^{13}C-linoleic acid treated midbrain neurons; (**B**, **D**, **F**, and **H**) ^{13}C-stearic acid treated midbrain neurons; (**A**-**C**) phosphatidylcholine (PC) headgroup $[C_5H_{15}PNO_4]^+$ at m/z 184.07; (**D**-**F**) ^{13}C-PC 30:1+K$^+$ at m/z 743.48; (**G**-**H**) ^{13}C-phosphatidylinositol (PI) fragment at m/z 420.26; and (**J**-**L**) ^{13}C-PI 38:7 at m/z 880.5. Peak intensity scale on the left of each image is from the lowest intensity (black) to highest (white), and scale bars are 100 µm. Fig **S5 C, F, I and L** compare the peak intensities of the imaged ions between the two treatment conditions. The peak intensity was binned and normalized to the total ion counts within the cell areas. Y-axis represents the normalized spectra, and the x-axis shows m/z. Error bars shown as standard error mean (SEM).

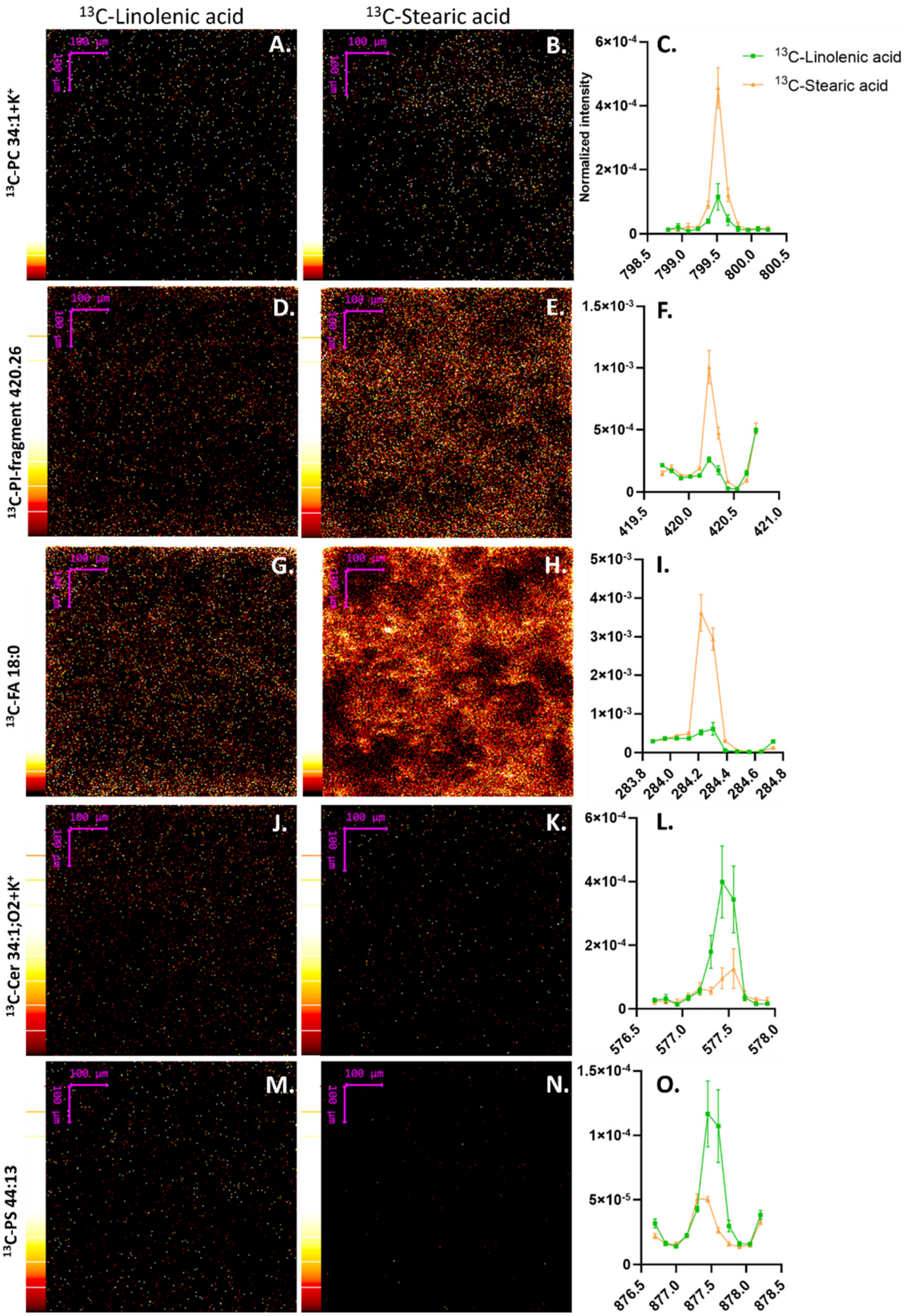

Figure S6. Comparison of plasma membrane lipid turnover in mature human midbrain neurons incubated with ^{13}C-linolenic acid and ^{13}C-stearic acid. SIMS ion images show the signal intensity of various lipids. (**A**-**C** and **J**-**L**) Positive mode; (**D**-**I** and **M**-**O**) negative mode. (**A**-**C**) ^{13}C-phosphatidylcholine (PC) 34:1+K^{+} at m/z 799.54; (**D**-**F**) ^{13}C-phosphatidylinositol (PI) fragment at m/z 420.26; (**G**-**I**) ^{13}C-fatty acid (FA) 18:0 (^{13}C-stearic acid) at m/z 284.26; (**J**-**L**) ^{13}C-

ceramide (Cer) 34:1;O_2+K^+ at m/z 577.48; and (**M**-**N**) ^{13}C-phosphatidylserine (PS) 44:13 at m/z 877.48. Peak intensity scale on the left of each image is from the lowest (black) to highest intensity (white) and scale bars are 100 µm. Fig **S6 C, F, I, L, and O** compare the peak intensities of the imaged ions between the two treatment conditions. The peak intensity was binned and normalized to the total ion counts within the cell areas. Y-axis represents the normalized spectra, and the x-axis shows m/z. Error bars shown as standard error mean (SEM).

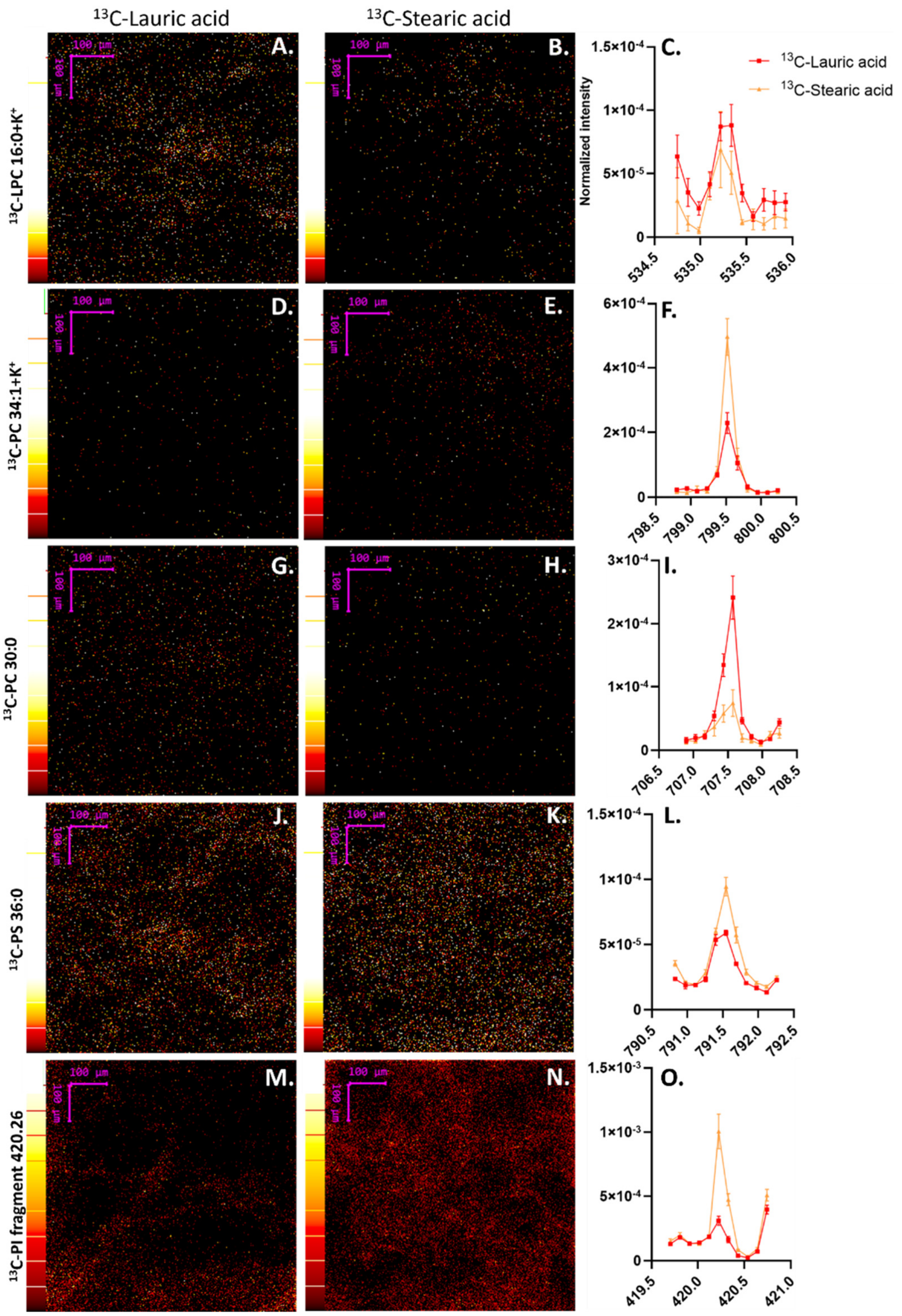

Figure S7. Comparison of plasma membrane lipid turnover in mature human midbrain neurons incubated with ^{13}C-lauric acid and ^{13}C-stearic acid. SIMS images at 256 x 256 pixels show the signal intensity of various lipids. (**A**-**I**) Positive mode; (**J**-**O**) negative mode; (**A**, **D**, **G**, **J**, and **M**) ^{13}C-lauric acid treated midbrain neurons; (**B**, **E**, **H**, **K**, and **N**) ^{13}C-stearic acid treated midbrain neurons; (**A**-**C**) ^{13}C-lysophosphatidylcholine (LPC) 16:0+K^+ at m/z at m/z 535.3; (**D**-**F**) ^{13}C-phosphatidylcholine (PC) 34:1+K^+ at m/z 799.54.; (**G**-**I**) ^{13}C-PC 30:0 at m/z 707.54; (**J**-**L**) ^{13}C-phosphatidylserine (PS) 36:0 at m/z 791.56 and (**M**-**O**) ^{13}C-phosphatidylinositol (PI) fragment at m/z 420.26. Peak intensity scale on the left of each image is from the lowest intensity (black) to highest (white), and scale bars are 100 µm. Fig **S7 C, F, I, L, and O** compare the peak intensities of the imaged ions between the two treatment conditions. The peak intensity was binned and normalized to the total ion counts within the cell areas. Y-axis represents the normalized spectra, and the x-axis shows m/z. Error bars shown as standard error mean (SEM).

Table S1. Summary of identified lipids in positive mode. Measured mass/charge (m/z) from ToF-SIMS analysis was compared to the reported m/z to delta calculate parts per million (Δppm). For calculation of Δppm the mass of the isotope was subtracted. Lipids identified with incorporated isotopes are marked as Yes (Y), which is included in the calculated formula m/z. The column *Assignment other than LMSD* refers to any other type of lipid assignment performed outside of using the LIPIDMAPS® LMSD database such as the LIPIDMAPS® computational database (COMP_DB) or from literature [12,16,67–270].

Measured m/z	Calculated m/z	Lipid name	Ion formula	Ionization	Δppm	Isotopic	Assignment other than LMSD
			Steryl ester (CE)				
570.56	570.53	CE 12:0	$C_{39}H_{69}O_2$	[M+H]+	60.33	Y	
608.63	608.58	CE 16:0	$C_{43}H_{75}O$	[M+H-H2O]+	80.22	Y	
			Ceramide (Cer)				
570.56	570.51	Cer 34:1;O4	$C_{34}H_{68}NO_5$	[M+H]+	92.22		
577.51	577.48	Cer 34:1;O2	$C_{34}H_{67}KNO_3$	[M+K]+	53.51	Y	
579.54	579.49	Cer 34:0;O2	$C_{34}H_{69}KNO_3$	[M+K]+	78.69	Y	
591.58	591.55	Cer 36:0;O2	$C_{36}H_{73}NNaO_3$	[M+Na]+	54.34	Y	
593.61	593.57	Cer 38:2;O2	$C_{38}H_{74}NO_3$	[M+H]+	76.11	Y	
600.62	600.57	Cer 38:1;O	$C_{38}H_{75}NNaO_2$	[M+Na]+	77.31		
601.6	601.57	Cer 38:1;O	$C_{38}H_{75}NNaO_2$	[M+Na]+	53.13	Y	
602.6	602.59	Cer 40:3;O	$C_{40}H_{76}NO_2$	[M+H]+	28.5		COMP_DB
608.62	608.63	Cer 40:0;O	$C_{40}H_{82}NO_2$	[M+H]+	-17.45		
613.52	613.53	Cer 38:3;O2	$C_{38}H_{71}NNaO_3$	[M+Na]+	-22.82	Y	
614.56	614.55	Cer 38:2;O2	$C_{38}H_{73}NNaO_3$	[M+Na]+	16.27		
630.61	630.62	Cer 40:0;O	$C_{40}H_{81}NNaO_2$	[M+Na]+	-8.5		
638.48	638.51	Cer 36:0;O4	$C_{36}H_{73}KNO_5$	[M+K]+	-55.91		
663.57	663.58	Cer 40:0;O2	$C_{40}H_{81}KNO_3$	[M+K]+	-15.1	Y	
666.6	666.64	Cer 42:1;O3	$C_{42}H_{84}NO_4$	[M+H]+	-60.23		
			Ceramide phosphoethanolamine (CerPE)				
654.46	654.46	CerPE 32:2;O2	$C_{34}H_{67}N_2NaO_6P$	[M+Na]+	0.29	Y	

658.53	658.49	CerPE 32:0;O2	$C_{34}H_{71}N_2NaO_6P$	[M+Na]+	57.09	Y	
762.56	762.56	CerPE 40:4;O2	$C_{42}H_{79}N_2NaO_6P$	[M+Na]+	3.48	Y	COMP_DB
764.54	764.57	CerPE 40:3;O2	$C_{42}H_{81}N_2NaO_6P$	[M+Na]+	-39.29	Y	
			Diacylglycerol (DG)				
551.51	551.5	DG 32:0	$C_{35}H_{67}O_4$	[M+H-H2O]+	5.39		
552.5	552.5	DG 32:0	$C_{35}H_{67}O_4$	[M+H-H2O]+	-6.06	Y	
573.49	573.49	DG 34:3	$C_{37}H_{65}O_4$	[M+H-H2O]+	-4.1		
577.52	577.52	DG 34:1	$C_{37}H_{69}O_4$	[M+H-H2O]+	-2.23		
602.49	602.52	DG 36:3	$C_{39}H_{69}O_4$	[M+H-H2O]+	-47.03	Y	
604.59	604.53	DG 36:2	$C_{39}H_{71}O_4$	[M+H-H2O]+	90.91	Y	
686.57	686.61	DG 42:3	$C_{45}H_{81}O_4$	[M+H-H2O]+	-62.44	Y	
			Simple Glc series (HexCer)				
673.57	673.54	HexCer 32:1;O2	$C_{38}H_{74}NO_8$	[M+H]+	44.61	Y	
680.5	680.45	HexCer 30:2;O2	$C_{36}H_{67}KNO_8$	[M+K]+	73.48		
682.47	682.47	HexCer 30:1;O2	$C_{36}H_{69}KNO_8$	[M+K]+	0.64		
708.54	708.5	HexCer 32:2;O3	$C_{38}H_{71}NNaO_9$	[M+Na]+	49.8		
			Monoacylglycerophosphocholine (LPC)				
505.25	505.25	LPC 14:1	$C_{22}H_{44}KNO_7P$	[M+K]+	0	Y	
505.33	505.34	LPC O-16:0	$C_{24}H_{52}NNaO_6P$	[M+Na]+	-19.83	Y	
535.31	535.3	LPC 16:0	$C_{24}H_{50}KNO_7P$	[M+K]+	30.19	Y	
659.51	659.48	LPC 26:0	$C_{34}H_{70}NNaO_7P$	[M+Na]+	45.56	Y	
			Monoacylglycerophosphoethanolamines (LPE)				
496.22	496.24	LPE 18:4	$C_{23}H_{40}NNaO_7P$	[M+Na]+	-52.66		
504.24	504.29	LPE 18:1	$C_{23}H_{46}NNaO_7P$	[M+Na]+	-97.85	Y	
			Monoacylglycerophosphoglycerols (LPG)				
503.24	503.24	LPG 18:5	$C_{24}H_{40}O_9P$	[M+H]+	0		COMP_DB
599.31	599.27	LPG 22:4	$C_{28}H_{49}KO_9P$	[M+K]+	52.7		
			Monoacylglycerol (MG)				
415.24	415.22	MG 20:5	$C_{23}H_{36}KO_4$	[M+K]+	47.03		
			Diacylglycerophosphocholine/phosphatidylcholine (PC)				
184.07	184.07	PC-headgroup	$C_5H_{15}PNO_4$	$[M+H]^+$	0		
185.07	185.07	PC-headgroup	$C_5H_{15}PNO_4$	$[M+H]^+$	0	Y	
478.33	478.35	PC 16:0 fragment			-41.81		Ref. 12
479.33	479.35	PC 16:0 fragment			-41.81	Y	Ref. 12
502.24	502.26	PC 16:4	$C_{24}H_{41}NO_8P$	[M+H]+	-29.41		
503.24	503.26	PC 16:4	$C_{24}H_{41}NO_8P$	[M+H]+	-30.18	Y	
507.24	507.29	PC 16:2	$C_{24}H_{45}NO_8P$	[M+H]+	-98.72	Y	Ref. 67, 68
509.26	509.3	PC 16:1	$C_{24}H_{47}NO_8P$	[M+H]+	-82.24	Y	
577.28	577.31	PC 18:0	$C_{26}H_{53}KNO_8P$	[M+K]+	-46.38	Y	
652.59	652.6	PC fragment 652.60		[M+Na]+	-12.18		Ref. 69
654.58	654.6	PC fragment 654.60		[M+Na]+	-33.72		Estimation based on ref. 69

682.39	682.39	PC 30:2-TMA	$C_{35}H_{63}KO_8P$	[M-TMA+K]+	0	Y	Estimation based on ref. 69
685.39	685.42	PC 30:0-TMA	$C_{35}H_{67}KO_8P$	[M-TMA+K]+	-43.77		
686.42	686.42	PC 30:0-TMA	$C_{35}H_{67}KO_8P$	[M-TMA+K]+	0	Y	
686.33	686.39	PC 32:6-TMA	$C_{37}H_{59}NaO_8P$	[M - TMA+Na]+	-87.54	Y	Estimation based on ref. 70
687.44	687.42	PC 26:1	$C_{34}H_{66}KNO_8P$	[M+K]+	32.09	Y	
688.38	688.4	PC 32:5-TMA	$C_{37}H_{61}NaO_8P$	[M - TMA+Na]+	-29.1	Y	Estimation based on ref. 70
698.46	698.48	PC 30:4	$C_{38}H_{69}NO_8P$	[M+H]+	-28.63		
707.5	707.54	PC 30:0	$C_{38}H_{77}NO_8P$	[M+H]+	-56.61	Y	
713.48	713.43	PC 28:2	$C_{36}H_{68}KNO_8P$	[M+K]+	71.36	Y	
713.45	713.45	PC 32:0-TMA	$C_{37}H_{71}KO_8P$	[M-TMA+K]+	0		Ref. 70
714.49	714.45	PC 32:0-TMA	$C_{37}H_{71}KO_8P$	[M-TMA+K]+	56.07	Y	Ref. 70
715.47	715.45	PC 28:1	$C_{36}H_{70}KNO_8P$	[M+K]+	31.3	Y	
717.43	717.46	PC 28:0	$C_{36}H_{72}KNO_8P$	[M+K]+	-39.51	Y	
725.51	725.49	PC 30:2	$C_{38}H_{72}NNaO_8P$	[M+Na]+	33.26	Y	
729.55	729.52	PC 30:0	$C_{38}H_{76}NNaO_8P$	[M+Na]+	43.03	Y	
732.55	732.55	PC 32:1	$C_{40}H_{79}NO_8P$	[M+H]+	-8.37		
734.57	734.57	PC 32:0	$C_{40}H_{81}NO_8P$	[M+H]+	-3.04		
735.55	735.57	PC 32:0	$C_{40}H_{81}NO_8P$	[M+H]+	-25.17	Y	
739.47	739.47	PC 34:1-TMA	$C_{39}H_{73}KO_8P$	[M-TMA+K]+	0		Ref. 16
740.51	740.47	PC 34:1-TMA	$C_{39}H_{73}KO_8P$	[M-TMA+K]+	54.09	Y	Ref. 16
741.51	741.46	PC 30:2	$C_{38}H_{72}KNO_8P$	[M+K]+	57.61	Y	
741.48	741.48	PC 34:0-TMA	$C_{39}H_{75}KO_8P$	[M-TMA+K]+	0		Ref. 16
742.48	742.48	PC 34:0-TMA	$C_{39}H_{75}KO_8P$	[M-TMA+K]+	0	Y	Ref. 16
743.54	743.48	PC 30:1	$C_{38}H_{74}NO_8PK$	[M+K]+	89.2	Y	
747.42	747.47	PC 32:5	$C_{40}H_{70}NNaO_8P$	[M+Na]+	-66.98	Y	
756.53	756.55	PC 32:0	$C_{40}H_{80}NNaO_8P$	[M+Na]+	-26.44		
760.58	760.59	PC 34:1	$C_{42}H_{83}NO_8P$	[M+H]+	-3.64		
761.56	761.59	PC 34:1	$C_{42}H_{83}NO_8P$	[M+H]+	-26.3	Y	
763.43	763.45	PC 32:5	$C_{40}H_{70}KNO_8P$	[M+K]+	-26.20	Y	
763.55	763.6	PC 34:0	$C_{42}H_{85}NO_8P$	[M+H]+	-69.66	Y	
769.53	769.49	PC 32:2	$C_{40}H_{76}KNO_8P$	[M+K]+	49.9	Y	
770.52	770.51	PC 32:1	$C_{40}H_{78}KNO_8P$	[M+K]+	9.76		
771.51	771.51	PC 32:1	$C_{40}H_{78}KNO_8P$	[M+K]+	5.88	Y	
772.53	772.53	PC 32:0	$C_{40}H_{80}KNO_8P$	[M+K]+	1.97		
773.52	773.53	PC 32:0	$C_{40}H_{80}KNO_8P$	[M+K]+	-13.1	Y	
789.63	789.62	PC 36:1	$C_{44}H_{87}NO_8P$	[M+H]+	13.25	Y	
798.55	798.54	PC 34:1	$C_{42}H_{82}KNO_8P$	[M+K]+	6.5		
799.52	799.54	PC 34:1	$C_{42}H_{82}KNO_8P$	[M+K]+	-27.07	Y	

800.54	800.56	PC 34:0	$C_{42}H_{84}KNO_8P$	[M+K]+	-19.49		
801.57	801.56	PC 34:0	$C_{42}H_{84}KNO_8P$	[M+K]+	10.56	Y	
806.52	806.57	PC 36:3	$C_{44}H_{82}NNaO_8P$	[M+Na]+	-61.99		
808.53	808.58	PC 36:2	$C_{44}H_{84}NNaO_8P$	[M+Na]+	-69.99		
812.55	812.62	PC 38:3	$C_{46}H_{87}NO_8P$	[M+H]+	-86.14		
814.59	814.63	PC 38:2	$C_{46}H_{89}NO_8P$	[M+H]+	-50.83		
820.52	820.53	PC 36:4	$C_{44}H_{80}KNO_8P$	$[M+K]^+$	-17		
824.55	824.56	PC 36:2	$C_{44}H_{84}KNO_8P$	[M+K]+	-5.68		
828.49	828.55	PC 38:6	$C_{46}H_{80}NNaO_8P$	[M+Na]+	-72.42		
829.51	829.55	PC 38:6	$C_{46}H_{80}NNaO_8P$	[M+Na]+	-48.28	Y	
829.59	829.59	PC 36:0	$C_{44}H_{88}KNO_8P$	[M+K]+	4.42	Y	
832.64	832.58	PC 38:4	$C_{46}H_{84}NNaO_8P$	[M+Na]+	72.07		
842.49	842.51	PC 38:7	$C_{46}H_{78}KNO_8P$	[M+K]+	-26.11		
846.48	846.54	PC 38:5	$C_{46}H_{82}KNO_8P$	[M+K]+	-75.45		
848.48	848.56	PC 38:4	$C_{46}H_{84}KNO_8P$	[M+K]+	-96.38		
868.45	868.53	PC 40:8	$C_{48}H_{80}KNO_8P$	[M+K]+	-86.87		
880.75	880.62	PC 40:2	$C_{48}H_{92}KNO_8P$	[M+K]+	-86.87		
892.47	892.53	PC 42:10	$C_{50}H_{80}KNO_8P$	[M+K]+	-65.82		
898.57	898.57	PC 42:7	$C_{50}H_{86}KNO_8P$	[M+K]+	0		
			Diacylglycerophosphoethanolamine/phosphatidylethanolamine (PE)				
547.3	547.32	PE 20:0	$C_{25}H_{50}NNaO_8P$	[M+Na]+	-36.61	Y	
684.42	684.46	PE 32:4	$C_{37}H_{67}NO_8P$	[M+H]+	-58.44	Y	
706.53	706.56	PE O-34:1	$C_{39}H_{79}NO_7P$	[M+H]+	-42.46	Y	
708.43	708.44	PE 32:4	$C_{37}H_{66}NNaO_8P$	[M+Na]+	-14.16	Y	
715.49	715.51	PE 34:3	$C_{39}H_{73}NO_8P$	[M+H]+	-16.43	Y	
734.55	734.59	PE O-36:1	$C_{41}H_{83}NO_7P$	[M+H]+	-54.45	Y	
744.55	744.55	PE 36:2	$C_{41}H_{79}NO_8P$	[M+H]+	0		
745.53	745.55	PE 36:2	$C_{41}H_{79}NO_8P$	[M+H]+	-26.86	Y	
756.53	756.48	PE 34:2	$C_{39}H_{74}KNO_8P$	[M+K]+	71.36	Y	
787.51	787.54	PE 36:0	$C_{41}H_{82}KNO_8P$	[M+K]+	-42.41	Y	
			Diacylglycerophosphoglycerol/phosphatidylglycerol (PG)				
575.28	575.3	PG 22:4	$C_{28}H_{48}O_{10}P$	[M+H]+	-34.76		COMP_DB
710.52	710.54	PG O-32:0	$C_{38}H_{78}O_9P$	[M+H]+	-26.09	Y	
745.55	746.5	PG 32:0	$C_{38}H_{75}NaO_{10}P$	[M+Na]+	64.17	Y	
746.55	746.5	PG 32:0	$C_{38}H_{75}NaO_{10}P$	[M+Na]+	64.17	Y	
774.6	774.53	PG 34:0	$C_{40}H_{79}NaO_{10}P$	[M+Na]+	90.49	Y	
784.56	784.55	PG O-38:5	$C_{44}H_{80}O_9P$	[M+H]+	11.06	Y	
800.55	800.55	PG 36:1	$C_{42}H_{81}NaO_{10}P$	[M+Na]+	4.78	Y	
802.54	802.56	PG 36:0	$C_{42}H_{83}NaO_{10}P$	[M+Na]+	-31.54	Y	
828.54	828.58	PG 40:4	$C_{46}H_{84}O_{10}P$	[M+H]+	-47.97	Y	
			Triacylglycerol (TG)				
732.55	732.56	TG 40:1	$C_{43}H_{80}KO_6$	[M+K]+	-12.3	Y	COMP_DB
762.61	762.61	TG 42:0	$C_{45}H_{86}KO_6$	[M+K]+	4.35	Y	
789.63	789.64	TG 44:0	$C_{47}H_{90}KO_6$	[M+K]+	-12.66		

References:

12. Agüi-Gonzalez, P.; Guobin, B.; Gomes De Castro, M.A.; Rizzoli, S.O.; Phan, N.T.N. Secondary Ion Mass Spectrometry Imaging Reveals Changes in the Lipid Structure of the Plasma Membranes of Hippocampal Neurons Following Drugs Affecting Neuronal Activity. ACS Chem Neurosci 2021, 12, 1542–1551, doi:10.1021/acschemneuro.1c00031/asset/images/large/cn1c00031_0004.jpeg.

16. Philipsen, M.H.; Phan, N.T.N.; Fletcher, J.S.; Ewing, A.G. Interplay between Cocaine, Drug Removal, and Methylphenidate Reversal on Phospholipid Alterations in Drosophila Brain Determined by Imaging Mass Spectrometry. ACS Chem Neurosci 2020, 11, doi:10.1021/acschemneuro.0c00014.

67. Xu, F.; Zou, L.; Lin, Q.; Ong, C.N. Use of Liquid Chromatography/Tandem Mass Spectrometry and Online Databases for Identification of Phosphocholines and Lysophosphatidylcholines in Human Red Blood Cells. Rapid Commun Mass Spectrom 2009, 23, 3243–3254, doi:10.1002/RCM.4246.

68. CHEBI:131441 - Phosphatidylcholine 16:2 Available online: https://www.ebi.ac.uk/chebi/CHEBI:131441 (accessed on 29 September 2025).

69. Konjevod, M.; Nedic Erjavec, G.; Nikolac Perkovic, M.; Sáiz, J.; Tudor, L.; Uzun, S.; Kozumplik, O.; Svob Strac, D.; Zarkovic, N.; Pivac, N. Metabolomics in Posttraumatic Stress Disorder: Untargeted Metabolomic Analysis of Plasma Samples from Croatian War Veterans. Free Radic Biol Med 2021, 162, 636–641, doi:10.1016/J.FREERADBIOMED.2020.11.024.

70. Philipsen, M.H.; Phan, N.T.N.; Fletcher, J.S.; Malmberg, P.; Ewing, A.G. Mass Spectrometry Imaging Shows Cocaine and Methylphenidate Have Opposite Effects on Major Lipids in Drosophila Brain. ACS Chem Neurosci 2018, 9, doi:10.1021/acschemneuro.8b00046.

Table S2. Summary of identified lipids in negative mode. Measured mass/charge (m/z) from ToF-SIMS analysis was compared to the reported m/z to delta calculate parts per million (Δppm). For calculation of Δppm the mass of the isotope was subtracted. Lipids identified with incorporated isotopes are marked as Yes (Y), which is included in the calculated formula m/z. The column *Assignment other than LMSD* refers to any other type of lipid assignment performed outside of using the LIPIDMAPS® LMSD database such as the LIPIDMAPS® computational database (COMP_DB) or from literature (71-74).

Measured m/z	Calculated m/z	Lipid name	Ion formula	Ionization	Δppm	Isotopic	Assignment other than LSMD
		1-(1Z-alkenyl),2-acylglycerophosphoethanolamine glycan (Am-Hex-PE)					
886.53	886.57	Am-Hex-PE O-36:5	$C_{47}H_{83}NO_{12}P$	[M-H]-	-40.1	Y	
			Steryl ester (CE)				
647.58	647.58	CE 18:2	$C_{45}H_{75}O_2$	[M-H]-	0.34		
670.57	670.56	CE 20:5	$C_{47}H_{73}O_2$	[M-H]-	15.74	Y	

			Ceramide (Cer)				
523.53	523.53	Cer 34:0;O	$C_{34}H_{68}NO_2$	[M-H]-	7.64	Y	
578.64	578.59	Cer 38:0;O	$C_{38}H_{76}NO_2$	[M-H]-	95.02		
638.6	638.61	Cer 40:0;O3	$C_{40}H_{80}NO_4$	[M-H]-	-9.04		
644.6	644.6	Cer 42:3;O2	$C_{42}H_{78}NO_3$	[M-H]-	-0.32		
			Ceramide phosphoethanolamine (CerPE)				
583.32	583.34	CerPE 28:6;O3	$C_{30}H_{50}N_2O_7P$	[M-H]-	-29.02	Y	COMP_DB
601.32	601.35	CerPE 28:5;O4	$C_{30}H_{52}N_2O_8P$	[M-H]-	-36.31	Y	COMP_DB
657.5	657.48	CerPE 34:3;O2	$C_{36}H_{68}N_2O_6P$	[M-H]-	34.45	Y	
673.48	673.48	CerPE 34:3;O3	$C_{36}H_{68}N_2O_7P$	[M-H]-	-1.56	Y	
695.48	695.52	CerPE 34:0;O4	$C_{36}H_{74}N_2O_8P$	[M-H]-	-59.84	Y	COMP_DB
705.51	705.54	CerPE 36:1;O3	$C_{38}H_{76}N_2O_7P$	[M-H]-	-35.62	Y	
707.49	707.56	CerPE 36:0;O3	$C_{38}H_{78}N_2O_7P$	[M-H]-	-93.97	Y	
715.55	715.56	CerPE 38:2;O2	$C_{40}H_{78}N_2O_6P$	[M-H]-	-8.7	Y	
723.5	723.49	CerPE 38:6;O3	$C_{40}H_{70}N_2O_7P$	[M-H]-	9.49	Y	COMP_DB
735.46	735.51	CerPE 36:2;O5	$C_{38}H_{74}N_2O_9P$	[M-H]-	-71.49	Y	COMP_DB
749.51	749.57	CerPE 38:1;O4	$C_{40}H_{80}N_2O_8P$	[M-H]-	-72.31	Y	COMP_DB
751.53	751.58	CerPE 38:0;O4	$C_{40}H_{82}N_2O_8P$	[M-H]-	-66.79	Y	COMP_DB
775.49	775.51	CerPE 38:4;O6	$C_{40}H_{74}N_2O_{10}P$	[M-H]-	-30.12	Y	COMP_DB
			Fatty acid (FA)				
237.08	237.08	FA 12:5;O3	$C_{12}H_{13}O_5$	[M-H]-	9.32		
253.21	253.22	FA 16:1	$C_{16}H_{29}O_2$	[M-H]-	-38.70		
255.22	255.23	FA 16:0	$C_{16}H_{31}O_2$	[M-H]-	-37.38		
256.23	256.23	FA 16:0	$C_{16}H_{31}O_2$	[M-H]-	-29.74	Y	
281.23	281.25	FA 18:1	$C_{18}H_{33}O_2$	[M-H]-	-50.45		
282.24	282.25	FA 18:1	$C_{18}H_{33}O_2$	[M-H]-	-30.03	Y	
283.25	283.26	FA 18:0	$C_{18}H_{35}O_2$	[M-H]-	-46.32		
284.26	284.26	FA 18:0	$C_{18}H_{35}O_2$	[M-H]-	-29.12	Y	
294.27	294.21	FA 18:3;O	$C_{18}H_{29}O_3$	[M-H]-	20.46	Y	
303.22	303.23	FA 20:4	$C_{20}H_{31}O_2$	[M-H]-	-37.59		
304.22	304.23	FA 20:4	$C_{20}H_{31}O_2$	[M-H]-	-26.84	Y	
305.24	305.25	FA 20:3	$C_{20}H_{33}O_2$	[M-H]-	-34.01		
331.26	331.26	FA 22:4	$C_{22}H_{35}O_2$	[M-H]-	-27.65		
534.58	534.53	FA 36:1	$C_{36}H_{69}O_2$	[M-H]-	97.72	Y	
			Simple Glc series (HexCer)				
670.56	670.53	HexCer 32:1;O2	$C_{38}H_{72}NO_8$	[M-H]-	48.53		
702.54	702.52	HexCer 32:1;O4	$C_{38}H_{72}NO_{10}$	[M-H]-	27.73		COMP_DB
703.51	703.52	HexCer 32:1;O4	$C_{38}H_{72}NO_{10}$	[M-H]-	-14.23	Y	
			Monoacylglycerophosphate/lysophosphatidic acid (LPA)				
311.16	311.16	LPA O-10:0	$C_{13}H_{28}O_6P$	[M-H]-	0		COMP_DB
408.21	408.22	LPA 16:1	$C_{19}H_{36}O_7P$	[M-H]-	-16.28	Y	
437.26	437.27	LPA 18:0	$C_{21}H_{42}O_7P$	[M-H]-	-9.28		
457.23	457.24	LPA 20:4	$C_{23}H_{38}O_7P$	[M-H]-	-6.93		
			Monoacylglycerophosphoethanolamine/lysophosphatidylethanolamine (LPE)				

474.28	474.26	LPE 18:3	$C_{23}H_{41}NO_7P$	[M-H]-	29.18		
582.31	582.34	LPE 26:6	$C_{31}H_{51}NO_7P$	[M-H]-	-56.93	Y	COMP_DB
Monoacylglycerophosphoglycerol/lysophosphatidylglycerol (LPG)							
502.23	502.23	LPG 18:5	$C_{24}H_{38}O_9P$	[M-H]-	3.06	Y	COMP_DB
504.23	504.24	LPG 18:4	$C_{24}H_{40}O_9P$	[M-H]-	-23.53	Y	
507.25	507.27	LPG 18:2	$C_{24}H_{44}O_9P$	[M-H]-	-37.79		
531.24	531.27	LPG 20:4	$C_{26}H_{44}O_9P$	[M-H]-	-67.99		
Monoacylglycerophosphoinositol/lysophosphatidylinositol (LPI)							
539.25	539.23	LPI 14:2	$C_{23}H_{40}O_{12}P$	[M-H]-	40.52		COMP_DB
542.25	542.24	LPI 14:1	$C_{23}H_{42}O_{12}P$	[M-H]-	17.52	Y	
581.29	581.31	LPI O-18:2	$C_{27}H_{50}O_{11}P$	[M-H]-	-32.82		
582.31	582.31	LPI O-18:2	$C_{27}H_{50}O_{11}P$	[M-H]-	1.59	Y	
583.3	583.33	LPI O-18:1	$C_{27}H_{52}O_{11}P$	[M-H]-	-42.94		
599.31	599.32	LPI 18:0	$C_{27}H_{52}O_{12}P$	[M-H]-	-21.99		
600.32	600.32	LPI 18:0	$C_{27}H_{52}O_{12}P$	[M-H]-	3.85	Y	
619.24	619.29	LPI 20:4	$C_{29}H_{48}O_{12}P$	[M-H]-	-83.24		
699.34	699.35	LPI 26:6	$C_{35}H_{56}O_{12}P$	[M-H]-	-15.85		COMP_DB
Monoacylglycerophosphoserine/lysophosphatidylserine (LPS)							
519.22	519.25	LPS 18:3	$C_{24}H_{41}NO_9P$	[M-H]-	-71.57	Y	
523.25	523.28	LPS 18:1	$C_{24}H_{45}NO_9P$	[M-H]-	-70.52	Y	
547.25	547.28	LPS 20:3	$C_{26}H_{45}NO_9P$	[M-H]-	-54.82	Y	
548.25	548.3	LPS 20:2	$C_{26}H_{47}NO_9P$	[M-H]-	-96.46		
Diacylglycerophosphate/phosphatidic acid (PA)							
579.3	579.29	PA 24:2;O3	$C_{27}H_{48}O_{11}P$	[M-H]-	10.2		
673.43	673.48	PA 34:1	$C_{37}H_{70}O_8P$	[M-H]-	-74.24		
687.55	687.53	PA O-36:1	$C_{39}H_{76}O_7P$	[M-H]-	23.26		
704.54	704.53	PA 36:0	$C_{39}H_{76}O_8P$	[M-H]-	11.37	Y	
Diacylglycerophosphoethanolamine/phosphatidylethanolamine (PE)							
600.32	600.35	PE 26:4	$C_{31}H_{53}NO_8P$	[M-H]-	-48.28	Y	COMP_DB
644.42	644.47	PE O-30:2	$C_{35}H_{67}NO_7P$	[M-H]-	-67.81		
708.47	708.46	PE 34:5	$C_{39}H_{67}NO_8P$	[M-H]-	15.55		
716.53	716.52	PE 34:1	$C_{39}H_{75}NO_8P$	[M-H]-	14.99		
718.54	718.54	PE 34:0	$C_{39}H_{77}NO_8P$	[M-H]-	-5.73		
722.5	722.51	PE O-36:5	$C_{41}H_{73}NO_7P$	[M-H]-	-18.75		
724.51	724.51	PE O-36:5	$C_{41}H_{73}NO_7P$	[M-H]-	0	Y	
726.52	726.54	PE O-36:3	$C_{41}H_{77}NO_7P$	[M-H]-	-39.34		
750.53	750.54	PE O-38:5	$C_{43}H_{77}NO_7P$	[M-H]-	-19.13		
752.53	752.54	PE O-38:5	$C_{43}H_{77}NO_7P$	[M-H]-	-16.39	Y	
806.46	806.53	PE 40:6;O	$C_{45}H_{77}NO_9P$	[M-H]-	-90.64		
858.51	858.55	PE 44:9;O	$C_{49}H_{79}NO_9P$	[M-H]-	-41.73	Y	COMP_DB
877.71	877.65	PE 46:5	$C_{51}H_{91}NO_8P$	[M-H]-	70.5	Y	COMP_DB
879.61	879.66	PE 46:4	$C_{51}H_{93}NO_8P$	[M-H]-	-58.74	Y	COMP_DB
Diacylglycerophosphoglycerol/phosphatidylglycerol (PG)							

553.27	553.31	PG 20:0	$C_{26}H_{50}O_{10}P$	[M-H]-	-85.79		
599.25	599.3	PG 24:5	$C_{30}H_{48}O_{10}P$	[M-H]-	-83.43		COMP_DB
721.49	721.5	PG 32:0	$C_{38}H_{74}O_{10}P$	[M-H]-	-11.23		
790.55	790.6	PG O-38:1	$C_{44}H_{86}O_{9}P$	[M-H]-	-69.03	Y	
Diacylglycerophosphoinositol/phosphatidylinositol (PI)							
241.0	241.01	PI headgroup	$C_{6}H_{10}PO_{8}$	[M-H]-	-41.79		Ref. 71
417.23	417.24	PI fragment 417.24	$C_{21}H_{38}O_{6}P$	[M-H]-	-35.17		Estimation based on Ref. 71
419.24	419.26	PI fragment 419.26	$C_{21}H_{40}O_{6}P$	[M-H]-	-42.22		Ref. 71
420.25	420.26	PI fragment 420.26	$C_{21}H_{40}O_{6}P$	[M-H]-	-18.37	Y	Ref. 71
780.48	780.47	PI 30:1	$C_{39}H_{72}O_{13}P$	[M-H]-	11.21	Y	
830.44	830.49	PI 34:4	$C_{43}H_{74}O_{13}P$	[M-H]-	-51.59	Y	
833.51	833.52	PI 34:2	$C_{43}H_{78}O_{13}P$	[M-H]-	-12.37		
836.52	836.53	PI 34:1	$C_{43}H_{80}O_{13}P$	[M-H]-	-20.92	Y	
852.46	852.47	PI 36:7	$C_{48}H_{72}NO_{10}P$	[M-H]-	-19.27	Y	
857.51	857.52	PI 36:4	$C_{45}H_{78}O_{13}P$	[M-H]-	-6.44		
859.53	859.53	PI 36:3	$C_{45}H_{80}O_{13}P$	[M-H]-	-3.07		
861.53	861.55	PI 36:2	$C_{45}H_{82}O_{13}P$	[M-H]-	-19.16		
876.47	876.47	PI 38:9	$C_{47}H_{72}O_{13}P$	[M-H]-	-3.08	Y	
878.5	878.49	PI 38:8	$C_{47}H_{74}O_{13}P$	[M-H]-	15.25	Y	
880.46	880.5	PI 38:7	$C_{47}H_{76}O_{13}P$	[M-H]-	-48.32	Y	
883.48	883.53	PI 38:5	$C_{47}H_{80}O_{13}P$	[M-H]-	-60.63		
885.54	885.55	PI 38:4	$C_{47}H_{82}O_{13}P$	[M-H]-	-13.78		
886.53	886.55	PI 38:4	$C_{47}H_{82}O_{13}P$	[M-H]-	-22.1	Y	
887.55	887.57	PI 38:3	$C_{47}H_{84}O_{13}P$	[M-H]-	-20.36		
888.55	888.57	PI 38:3	$C_{47}H_{84}O_{13}P$	[M-H]-	-13.76	Y	
889.59	889.58	PI 38:2	$C_{47}H_{86}O_{13}P$	[M-H]-	12.4		
890.59	890.58	PI 38:2	$C_{47}H_{86}O_{13}P$	[M-H]-	13.1	Y	
901.63	901.62	PI O-40:3	$C_{49}H_{90}O_{12}P$	[M-H]-	11.34		
Diacylglycerophosphoserine/phosphatidylserine (PS)							
581.29	581.29	PS 20:1;O	$C_{26}H_{47}NO_{11}P$	[M-H]-	-2.57	Y	
582.3	582.3	PS 20:0;O	$C_{26}H_{49}NO_{11}P$	[M-H]-	0.62		COMP_DB
606.32	606.3	PS 22:2;O	$C_{28}H_{49}NO_{11}P$	[M-H]-	26.19		
699.45	699.5	PS 36:2 fragment	$C_{39}H_{72}O_{8}P$	[M-87-H]-	-66.45		Ref. 73
701.5	701.51	PS 36:1 fragment	$C_{39}H_{74}O_{8}P$	[M-87-H]-	-14.25		Ref. 74
702.54	702.51	PS 36:1 fragment	$C_{39}H_{74}O_{8}P$	[M-87-H]-	42.76	Y	Ref. 74
748.51	748.55	PS O-34:0	$C_{40}H_{79}NO_{9}P$	[M-H]-	-50.84		
751.53	751.53	PS 40:4 fragment	$C_{43}H_{76}O_{8}P$	[M-87-H]-	2.95		Estimation based on ref. 72 and 73
788.53	788.54	PS 36:1	$C_{42}H_{79}NO_{10}P$	[M-H]-	-23.0		
791.53	791.56	PS 36:0	$C_{42}H_{81}NO_{10}P$	[M-H]-	-41.62	Y	
800.56	800.58	PS O-38:2	$C_{44}H_{83}NO_{9}P$	[M-H]-	-24.98		

810.48	810.53	PS 38:4	$C_{44}H_{77}NO_{10}P$	[M-H]-	-66.19		
834.51	834.53	PS 40:6	$C_{46}H_{77}NO_{10}P$	[M-H]-	-22.89		
861.5	861.54	PS 42:7	$C_{48}H_{79}NO_{10}P$	[M-H]-	-47.68	Y	
868.56	868.61	PS 42:3	$C_{48}H_{87}NO_{10}P$	[M-H]-	-50.17		
875.46	875.47	PS 44:14	$C_{50}H_{69}NO_{10}P$	[M-H]-	-4.52	Y	COMP_DB
877.47	877.48	PS 44:13	$C_{50}H_{71}NO_{10}P$	[M-H]-	-13.27	Y	COMP_DB
879.46	879.5	PS 44:12	$C_{50}H_{73}NO_{10}P$	[M-H]-	-39.43	Y	
881.48	881.51	PS 44:11	$C_{50}H_{74}NO_{10}P$	[M-H]-	-41.04	Y	COMP_DB
884.53	884.54	PS 44:9	$C_{50}H_{79}NO_{10}P$	[M-H]-	-8.78		COMP_DB
885.56	885.54	PS 44:9	$C_{50}H_{79}NO_{10}P$	[M-H]-	13.93	Y	COMP_DB
886.56	886.56	PS 44:8	$C_{50}H_{81}NO_{10}P$	[M-H]-	1.08		
887.57	887.56	PS 44:8	$C_{50}H_{81}NO_{10}P$	[M-H]-	11.43	Y	
898.6	898.65	PS 44:2	$C_{50}H_{93}NO_{10}P$	[M-H]-	-57.42		
899.65	899.65	PS 44:2	$C_{50}H_{93}NO_{10}P$	[M-H]-	-2.46	Y	
903.64	903.69	PS 44:0	$C_{50}H_{97}NO_{10}P$	[M-H]-	-54.79	Y	
Sulfoglycosphingolipid/sulfatide (SHexCer)							
798.47	798.48	SHexCer 36:5;O2	$C_{42}H_{72}NO_{11}S$	[M-H]-	-22.71		COMP_DB
888.56	888.62	SHexCer 42:2;O2	$C_{48}H_{90}NO_{11}S$	[M-H]-	-67.52		
889.59	889.62	SHexCer 42:2;O2	$C_{48}H_{90}NO_{11}S$	[M-H]-	-35.75	Y	
904.59	904.62	SHexCer 42:2;O3	$C_{48}H_{90}NO_{12}S$	[M-H]-	-29.65		
906.64	906.63	SHexCer 42:1;O3	$C_{48}H_{92}NO_{12}S$	[M-H]-	1.74		
908.63	908.65	SHexCer 42:0;O3	$C_{48}H_{94}NO_{12}S$	[M-H]-	-17.88		COMP_DB
912.61	912.62	SHexCer 44:4;O2	$C_{50}H_{90}NO_{11}S$	[M-H]-	-18.58		COMP_DB
914.62	914.64	SHexCer 44:3;O2	$C_{50}H_{92}NO_{11}S$	[M-H]-	-22.22		COMP_DB
916.63	916.66	SHexCer 44:2;O2	$C_{50}H_{94}NO_{11}S$	[M-H]-	-32.02		
Cholesterol and derivatives/sterol (ST)							
473.27	473.29	ST 28:4;O6	$C_{28}H_{41}O_6$	[M-H]-	-42.26		
Triacylglycerol (TG)							
861.82	861.79	TG 52:0	$C_{55}H_{105}O_6$	[M-H]-	32.87		

References:

71. Hsu, F.F.; Turk, J. Characterization of Phosphatidylinositol, Phosphatidylinositol-4-Phosphate, and Phosphatidylinositol-4,5-Bisphosphate by Electrospray Ionization Tandem Mass Spectrometry: A Mechanistic Study. J Am Soc Mass Spectrom 2000, 11, 986–999, doi:10.1016/S1044-0305(00)00172-0.

72. Saud, Z.; Tyrrell, V.J.; Zaragkoulias, A.; Protty, M.B.; Statkute, E.; Rubina, A.; Bentley, K.; White, D.A.; Dos Santos Rodrigues, P.; Murphy, R.C.; et al. The SARS-CoV2 Envelope Differs from Host Cells, Exposes Procoagulant Li-pids, and Is Disrupted in Vivo by Oral Rinses. J Lipid Res 2022, 63, 100208, doi:10.1016/J.JLR.2022.100208.

73. Jackson, S.N.; Wang, H.Y.J.; Woods, A.S. In Situ Structural Characterization of Glycerophospholipids and Sulfatides in Brain Tissue Using MALDI-MS/MS. J Am Soc Mass Spectrom 2007, 18, 17–26, doi:10.1016/J.JASMS.2006.08.015.

74. Bakker, B.; Eijkel, G.B.; Heeren, R.M.A.; Karperien, M.; Post, J.N.; Cillero-Pastor, B. Oxygen-Dependent Lipid Profiles of Three-Dimensional Cultured Human Chondrocytes Revealed by MALDI-MSI. Anal Chem 2017, 89, 9438–9444, doi:10.1021/acs.analchem.7b02265/suppl_file/ac7b02265_si_001.pdf.
